# Genetic architecture of epigenetic cortical clock age in brain tissue from older individuals: alterations in *CD46* and other loci

**DOI:** 10.1080/15592294.2024.2392050

**Published:** 2024-08-22

**Authors:** Francine Grodstein, Bernardo Lemos, Jingyun Yang, Katia de Paiva Lopes, Ricardo A. Vialle, Nicholas Seyfried, Yanling Wang, Gemma Shireby, Eilis Hannon, Alan Thomas, Keeley Brookes, Jonathan Mill, Philip L. De Jager, David A. Bennett

**Affiliations:** a Rush Alzheimer’s Disease Center, Rush University Medical Center, Chicago, IL, USA; b Department of Internal Medicine, Rush University Medical Center, Chicago, IL, USA; c Coit Center for Longevity and Neurotherapeutics, Department of Pharmacology and Toxicology, R. Ken Coit College of Pharmacy, The University of Arizona, Tucson, AZ, USA; d Department of Neurological Sciences, Rush University Medical Center, Chicago, IL, USA; e Department of Biochemistry, and Center for Neurodegenerative Diseases, Emory University, Atlanta, GA, USA; f Department of Clinical and Biomedical Sciences, University of Exeter Medical School, University of Exeter, Exeter, UK; g Translational and Clinical Research Institute, Newcastle University, Newcastle Upon Tyne, UK; h Biosciences, School of Science and Technology, Nottingham Trent University, Nottingham, UK; i Center for Translational and Computational Neuroimmunology, Department of Neurology, and Taub Institute for Research on Alzheimer’s Disease and the Aging Brain, Columbia University Irving Medical Center, New York, NY, USA

**Keywords:** Aging, epigenetics, brain, dementia

## Abstract

The cortical epigenetic clock was developed in brain tissue as a biomarker of brain aging. As one way to identify mechanisms underlying aging, we conducted a GWAS of cortical age. We leveraged postmortem cortex tissue and genotyping array data from 694 participants of the Rush Memory and Aging Project and Religious Orders Study (ROSMAP; 11000,000 SNPs), and meta-analysed ROSMAP with 522 participants of Brains for Dementia Research (5,000,000 overlapping SNPs). We confirmed results using eQTL (cortical bulk and single nucleus gene expression), cortical protein levels (ROSMAP), and phenome-wide association studies (clinical/neuropathologic phenotypes, ROSMAP). In the meta-analysis, the strongest association was rs4244620 (*p* = 1.29 × 10^−7^), which also exhibited FDR-significant cis-eQTL effects for *CD46* in bulk and single nucleus (microglia, astrocyte, oligodendrocyte, neuron) cortical gene expression. Additionally, rs4244620 was nominally associated with lower cognition, faster slopes of cognitive decline, and greater Parkinsonian signs (n ~ 1700 ROSMAP with SNP/phenotypic data; all *p* ≤ 0.04). In ROSMAP alone, the top SNP was rs4721030 (*p* = 8.64 × 10^−8^) annotated to *TMEM106B* and *THSD7A*. Further, in ROSMAP (*n* = 849), TMEM106B and THSD7A protein levels in cortex were related to many phenotypes, including greater AD pathology and lower cognition (all *p* ≤ 0.0007). Overall, we identified converging evidence of *CD46* and possibly *TMEM106B/THSD7A* for potential roles in cortical epigenetic clock age.

## Introduction

Biomarkers of aging are critical tools for developing and testing interventions to delay aging-associated declines in health, as well as for assessing mechanisms underlying aging processes. Given the high prevalence and burden of dementia in older populations, biomarkers of brain aging have public health relevance [1].

Epigenetic clocks are among the most successful biomarkers of aging to date; these clocks use DNA methylation (DNAm) states across the genome to yield a ‘signature’ of aging, trained against chronological age or other proxies for health status [2]. Recently, a cortical epigenetic clock was developed, specifically using DNAm states in brain tissue [3]. This clock integrates 347 CpG sites into an ‘aging score’ and performs particularly well in distinguishing neurodegenerative phenotypes compared to clocks designed in blood or other tissues [3]. Identifying genes regulating cortical clock age is one pathway to discovering novel mechanisms underlying brain aging (although other factors which may be related to epigenetic age, such as environmental exposures, will be important to examine as well).

In our previous research, we calculated cortical clock age in postmortem prefrontal cortex specimens from deceased participants of the Religious Orders Study and Rush Memory and Aging Project, all of whom had been followed during life with annual clinical exams [4,5]. We observed strong associations of cortical clock age with multiple clinical and pathologic dementia-related phenotypes, as well as other aging-related phenotypes. Thus, to begin to understand the genetic architecture of cortical clock – which is one approach to potentially identify mechanisms at the intersection of aging and neurodegeneration – we conducted a genome-wide association study (GWAS) of cortical clock age in three large cohorts of aging, all with cortical brain specimens, available DNA methylation array data, and genome-wide array data.

## Methods

### Study Populations

Since epigenetic clock aging appears to differ in older than in younger persons [3,6], we focused our work here in three cohorts which primarily include older participants: Religious Orders Study (ROS), Rush Memory and Aging Project (MAP), and Brains for Dementia Research (BDR). The Religious Orders Study was initiated in 1994 [7] and includes older priests, nuns, and brothers from across the US, free of known dementia at the time of enrolment. Participants agreed to annual neurological exams, neuropsychological testing, and blood draw. Over 1,500 participants completed a baseline evaluation as of November 2023. The follow-up rate and autopsies exceed 90%. The Rush Memory and Aging Project was established in 1997 [7], with virtually identical design and data collection, and includes older men and women from across the Chicago metropolitan area, without known dementia at enrolment; over 2,300 participants completed a baseline evaluation as of November 2023. The follow-up rate exceeds 90% and the autopsy rate exceeds 80%. Both studies were approved by an Institutional Review Board of Rush University Medical Center. All participants signed an informed consent and Anatomical Gift Act for organ donation. More than 2000 autopsies have been obtained to date. The Brains for Dementia Research brain bank was established in 2008 in the United Kingdom, as a research tissue bank across six dementia research centres, with uniform procedures [8]. Participants were recruited from the community and provided informed consent for regular assessment and their consent to donate their brain for research upon death. At the time, these analyses were initiated, over 600 postmortem brains were available for research. The study was approved by the National Research Ethics Service.

### Assessment of DNA methylation states and Epigenetic Clock Age

In ROSMAP brain specimens, DNA methylation was measured in tissue from the dorsolateral prefrontal cortex (DLPFC). Briefly, 100 mg frozen sections were thawed on ice, with the grey matter dissected from the white matter, as previously described in detail [9]. The Qiagen QIAamp DNA mini protocol was used for DNA isolation. DNA methylation profiles were generated using the Illumina Infinium HumanMethylation450 platform. In more recent work with these specimens, processing methods were updated compared to our previous publications [9]. The raw signal intensities were imported into the R statistical environment with functions from the methylumi package and further processed with the wateRmelon [10] package. Initial quality control assessment was performed using functions in the methylumi package to exclude samples with inefficient bisulfite conversion (<90%) as well as outliers. Further preprocessing was conducted using the wateRmelon package by applying a p-filter. Probes having more than 1% of samples with a detection p-value greater than 0.05 and a beadcount lower than 3 in more than 5% of samples were excluded. Finally, the filtered data were normalized with ‘dasen.’ Non-CpG SNP (single nucleotide polymorphism) probes, probes that had been reported to contain common (MAF > 5%) SNPs in the CG or single base extension position or probes that were non-specific or mismapped, were flagged and disregarded in the evaluation of our results. The resulting dataset for analysis here consisted of 730 samples with 423,841 probes each. At each probe, DNAm level was represented as a beta value, that is, the ratio of the methylated probe intensity to the sum of methylated and unmethylated probe intensities. The values ranged from 0 to 1, where a larger value indicates higher methylation.

In BDR, DNA was isolated from prefrontal cortex samples using the Qiagen AllPrep DNA/RNA 96 kit [3]. Genome-wide DNA methylation was profiled using the Illumina EPIC DNAm array, which interrogates >850,000 DNA methylation sites. All work was done in the R statistical environment, and processing used either wateRmelon or bigmelon packages. Briefly, the quality control pipeline included (i) checking signal intensities and excluding poorly performing samples, (ii) calculating a bisulphite conversion statistic for each sample, excluding those with a conversion rate <80%, (iii) using p-filter function to exclude samples with >1% of probes with a detection p-value >0.05 and probes with >1% of samples with detection p-value >0.05, and (iv) removing cross-hybridizing and SNP probes. The filtered data were normalized with ‘dasen.’ This resulted in 800,916 DNA methylation sites in 610 prefrontal cortex specimens.

The cortical clock was calculated using publicly available code (https://github.com/gemmashireby/CorticalCloc). Briefly, the original cortical clock was trained in cortex tissue, specifically including large samples of older participants, to focus on brain aging. The clock was trained only using 383,547 CpG sites that were common across the Illumina 450K and EPIC arrays. The cortical clock was trained against chronological age, and integrates 347 CpG sites into a brain age score.

### Assessment of genome-wide array Data.

For the ROSMAP participants, genotyping was performed on either the Affymetrix GeneChip 6.0 platform (1,878 participants, 909,600 SNPs) or the Illumina OmniQuad Express platform (456 participants, 730,525 SNPs). DNA was extracted from whole blood, lymphocytes, or frozen brain tissue, as previously described [11]. To minimize population admixture, only self-declared non-Latino individuals of European ancestry were genotyped. Then, genotyping data from both platforms were processed using PLINK software, version 1.08p [12], with standard quality control (QC) metrics such as genotype success rate *>* 0.95, Hardy-Weinberg equilibrium *p >*0.001, and mishap test *<* 1 × 10 − 9, as previously described [11,13]. EIGENSTRAT was used with default settings to remove population outliers and to generate a genotype covariance matrix [14], and closely related participants were removed. After these QC steps, 1,709 individuals and 750,173 autosomal markers from the Affymetrix GeneChip 6.0 platform, and 382 individuals and 627,742 autosomal markers from the Illumina OmniQuad Express platform were used for imputation. Dosages for SNPs (*>*35 million) were imputed on the haplotype reference consortium (HRC) panel. Analyses filtered SNPs based on minor allele frequency (MAF) *>* 0.01 and imputation INFO score *>* 0.3. This yielded 11,507,242 SNPs for analysis.

For BDR participants, DNA was extracted from brain tissue, and the NeuroChip was used, a custom Illumina genotyping array with an extensive genome-wide backbone (*n* = 306,670 variants) and a custom content covering 179,467 variants specific to neurological diseases [15]. The quality control of the NeuroChip was completed in GenomeStudio (version 2.0, Illumina) and PLINK v1.9 [12]. Manual curation of SNP clustering performed by Genome Studio algorithms was conducted on all SNPs. SNPs with ambiguous clustering of the three genotypes were removed. Likewise, individual sample signals which lie ambiguously between genotype clusters were also removed. Average GenTrain score, cluster separation and SNP call frequency were 0.83, 0.85 and 0.996, respectively, in the exported PLINK compatible files. Genotype data was aligned to the GRCh37/hg19 reference genome. SNPs with a minor allele frequency of less than 1%, had genotype calls of less than 95% and had control samples that significantly deviated from Hardy-Weinberg Equilibrium (*p* < 0.0001) were removed. Imputation was done using the 1000 Genomes Project reference panel and yielded 6,607,832 SNPs for analysis here.

Across the ROSMAP and BDR datasets, there were 5,091,857 SNPs in common.

### Genome-wide association analyses

We performed GWAS on cortical clock age in DLPFC in ROSMAP, using linear regression models with cortical clock age as the dependent variable and genotype as the independent variable; covariates included age at death, sex, GWAS chip, study, and the top three principal components derived from the genetic covariance matrix. Association testing was done using an additive model for dose. Due to our modest sample size, for statistical significance, we used a suggestive threshold of *p* < 10^−5^, as has been done previously [16]. We also removed SNPs if the beta estimate was greater than 30, as has been done in previous GWAS of epigenetic clocks [17], since such extreme values are not highly plausible.

We conducted a similar GWAS in the BDR data and meta-analysed results from ROSMAP and BDR, limited to the common SNPs across these cohorts; then, we used METAL to conduct fixed-effect meta-analysis combining results across the cohorts [18]. The larger sample size in this meta-analysis has important advantages in terms of the stability of findings. For all GWAS, we calculated lambda values and created Q–Q plots of the -log10 p-values.

In sensitivity analyses, since cell proportions in bulk tissue can be related to aging and DNAm, we also considered potential confounding by neuron proportion. We estimated the neuron proportion using the CETS algorithm [19], which utilizes DNAm states from the Illumina array. We added neuron proportion as a covariate to our models of SNPs and cortical clock age. In additional sensitivity analyses, we controlled for seven cell-type proportions (i.e., inhibitory neurons, excitatory neurons, astrocytes, endothelial cells, oligodendrocytes, oligodendrocyte precursor cells, microglia), using single-cell methylation sequencing as the reference with the Houseman deconvolution algorithm [20]. These were sensitivity analyses because we have consistently found in our previous DNAm research in brain tissue that control for cell-type proportion does not meaningfully change findings [4,5,9].

### Clumping analysis

To assess the independent loci associated with clock age, we used PLINK to clump SNPs within the suggestive loci (250 kb), setting the linkage disequilibrium (LD) r^2^ >0.5 as a threshold [12]. The SNP with the most significant relation to clock age within each independent locus was selected as the leading SNP for that locus.

### Functional annotation

We used FUMA (Functional Mapping and Annotation of Genome-wide Association Studies) [21] to examine the genetic foundations of cortical clock age. Specifically, we utilized the FUMA SNP2GENE function to annotate SNPs and map them to their respective genes, focusing on their biological functions. Our approach included positional mapping with a maximum distance of 10 kb from each SNP. We set the P-value threshold for lead SNPs at 10^−5^. Apart from these specific settings, we adhered to the standard configurations in SNP2GENE, such as an r^2^ threshold of 0.6 for identifying independent significant SNPs.

### Expression quantitative trait loci (eQTL) and colocalization analyses

We queried eQTL data from BrainMeta [22]. This included eQTL mapping using RNA-seq data comprising 2,865 brain cortex samples obtained from 2,443 unrelated individuals of European ancestry with genome-wide SNP data. These RNA-seq data were sourced from seven cohorts, including ROSMAP.

In addition to considering eQTL in BrainMeta, we also conducted colocalization analyses to test if lead SNPs from either the ROSMAP GWAS or the ROSMAP/BDR meta-analysis could be linked to epigenetic age by dysregulation of gene expression. Specifically, we considered our GWAS hits in the context of the bulk RNA-Seq from cortex along with publicly available eQTL and splicing QTL (sQTL) from the Myeloid Cells in Neurodegenerative Diseases (MyND) [23] and Microglia Genomic Atlas (MiGA) [24] datasets of gene expression. For colocalization analyses, we used the COLOC package from Giambartolomei et al. [25], with default parameters. Our criteria for considering a signal as colocalized was posterior probability of 0.8 or higher.

In further analyses, we also used single nucleus RNA-Seq profiling available in 424 of the ROSMAP DLPFC specimens as a way to better understand possible biologic pathways [24]; this included eQTLs from seven cell types (astrocytes, endothelial cells, excitatory neurons, inhibitory neurons, microglia, oligodendrocytes, and oligodendrocyte precursor cells [OPC]). Briefly, grey matter was processed in batches, and 5000 nuclei from each batch were pooled, prepared, and sequenced using either Illumina HiSeqX at the Broad Institute’s Genomics Platform or Illumina NovaSeq 6000 at the New York Genome Center, with a target coverage of 1 million reads per channel [26,27]. The data were first processed using the CellRanger software (v6.0.0; 10× Genomics) and quality controlled. Each nucleus was assigned back to its participants based on its genotypes. Cell types of nuclei were determined, and pseudo-bulk matrices were created by summing counts per individual. Within each cell type, genes with CPM > 1 in 80% of samples were retained and normalized using *tmm.voom*.

Finally, we also considered colocalization across the GWAS results for lead SNPs with seven cell types from the ROSMAP snRNA-Seq to identify common causal variants for cortical clock age and cell-type gene expression.

### Functional enrichment analyses

We used the platform snpXplorer to conduct functional analyses [28]. This included GO enrichment of biological processes and KEGG pathways, as well as an examination of any overlap of the SNPs and genes identified in our GWAS with previous associations reported in the GWAS catalog. We conducted two separate sets of functional analyses, one for the results of the ROSMAP GWAS and one for the meta-analysis of ROSMAP and BDR.

### Analyses of aging phenotypes

As an additional approach to understand how SNPs of interest was related to function, we also examined the lead SNPs in relation to aging phenotypes available in ROSMAP. We first considered common postmortem neuropathologies, available in 1723 participants who also had SNP array data. Due to the broad range of SNP frequencies, including some with fairly low prevalence, we focused on the most common neuropathologies. This included AD pathologies (global Alzheimer’s disease pathology burden, amyloid-β load, PHFtau tangle density) and cerebrovascular pathologies (atherosclerosis and arteriolosclerosis, the two most common cerebrovascular pathologies in the ROSMAP cohorts); these neuropathologies are described in detail elsewhere [5,29]. In addition to neuropathologies, we also examined lead SNPs in relation to key clinical phenotypes, available in 1885 participants from annual clinical assessments prior to death; this included two cognitive phenotypes and two motor phenotypes, described in detail elsewhere [5]. Specifically, we examined the following: (i) global cognitive function at cohort baseline (the average of 17 cognitive tasks, such that higher scores represent better function), and slopes of cognitive decline from baseline to death; (ii) diagnosis of dementia as of death; (iii) motor function at baseline and over time (a combined score across 10 tests, such as grip strength and timed walking, such that higher scores represent better function); and (iv) Parkinsonian signs (the average of four domains of the 26-item modified United Parkinson’s Disease Rating Scale: bradykinesia, gait, rigidity, tremor, such that higher scores represent worse function).

Finally, we also had data available on proteins in DLPFC among 849 participants. Thus, as a further way to confirm functionality, we examined relations of relevant proteins to these same aging phenotypes (using similar methods as described above). Specifically, based on the gene annotation described above, we identified corresponding proteins for the leading SNPs, when available. The proteins were measured using a multiplex mass spectrometry-based proteomics approach with tandem mass tag (TMTs) to analyse frozen tissue samples in the DLPFC. Briefly, 100 mg frozen sections were thawed on ice, with the grey matter dissected from the white matter, as previously described in detail [30]. Details of the mass spectrometry – based proteomics, database searches, and quality control have been previously described [30]. To summarize, the samples were homogenized, and the protein concentration was determined. After protein digestion, isobaric TMT peptide labelling and high pH fractionation were performed. Fractions were then analysed by liquid chromatography-mass spectrometry. The resulting mass spectrometry spectra were searched against the UniProt human protein database, with individual protein abundance checked against the global internal standard. An additional data process included regressing out technical confounders. A total of 8425 proteins passed the final quality control.

In all these analyses of aging phenotypes, for continuous phenotypes measured at death (e.g., global AD pathology), we used linear regression models; for continuous phenotypes measured repeatedly over time (e.g., global cognition), we used generalized linear mixed models. For categorical phenotypes (e.g., atherosclerosis severity, dementia diagnosis as of death), we used logistic regression models. All models controlled for age at death, sex, education, and cohort.

**Figure 1.**
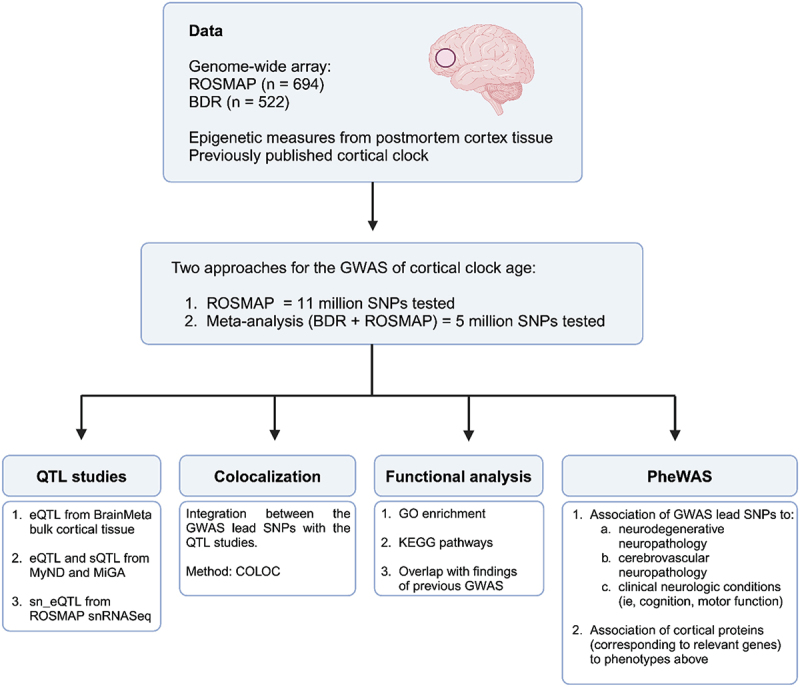
Flowchart of study design.

A flow chart summarizing our overall analytic approach is presented in Figure 1.

### Data availability

ROSMAP resources can be requested at https://www.radc.rush.edu and www.synapse.org.

The BDR DNA methylation data have been deposited in the Dementias Platform UK (DPUK) data portal (https://portal.dementiasplatform.uk/CohortDirectory/Item?fingerPrintID=BDR) and the Gene Expression Omnibus (GEO) at accession number GSE197305.

## Results

The 694 ROSMAP participants included in our GWAS had a mean age at death of 88 y; the mean cortical clock age was 86.5 (SD 6.7) y, slightly younger than the chronological age (Table 1). Approximately one-third of the ROSMAP sample was male, and participants were split fairly evenly across the ROS and MAP cohorts. Mean education was 16 y. Approximately 50% had pathologic AD, while 60% had clinical diagnosis of dementia as of death. The BDR cohort (*n* = 522) had a mean age at death of 83 (SD 9.0) y, and the mean cortical clock age was 84 y. Approximately half of the cohort was male, and mean education was approximately 13 y. Nearly half of the participants had pathologic AD, and about 60% had clinical dementia.

**Table 1.** Characteristics of study cohorts.

Characteristic	Religious Orders Study/Rush Memory and Aging Project (*n* = 694) Mean (SD) or %	Brains for Dementia Research (*n* = 522) Mean (SD) or %
Mean age at death	88.1 (6.7)	83.4 (9.0)
Mean cortical clock age	86.5 (6.0)	84.0 (7.2)
Male	37%	53%
Mean years education	16.4 (3.6)	12.5 (3.5)*
Memory and Aging Project	46%	n/a
Religious Orders Study	54%	n/a
Pathologic Alzheimer’s disease	62%	43%
Clinical dementia	58%	59%*

*Educational attainment was reported by 369 participants in the Brains for Dementia Research cohort, and clinical dementia status was known in 443 participants.

### GWAS of cortical clock age

**Figure 2.**
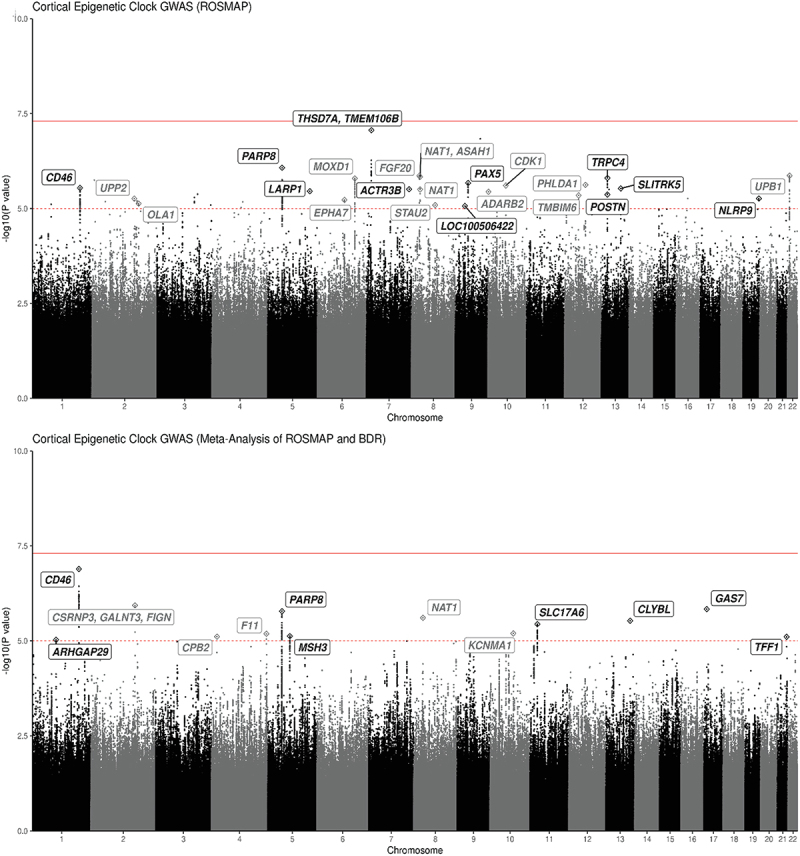
Manhattan plot: GWAS of cortical clock age for ROSMAP and Meta-analysis of ROSMAP/BDR.

In our primary analyses, we used fixed-effect meta-analysis to combine the ROSMAP and BDR GWAS results, limited to the 5 million SNPs which were common after imputation to the Affymetrix GeneChip 6.0 and Illumina OmniQuad Express chip (from ROSMAP) as well as the NeuroChip custom array from BDR. We found 110 SNPs which met our suggestive criteria for statistical significance of *p* < 10^−5^ (Figure 2 and eTable 1). We found no evidence of genomic inflation for this meta-analysis (eFigure 1). After excluding SNPs based on the frequency of effect allele, there were 13 independent loci; Table 2 lists the 13 leading SNPs. The strongest association was for rs4244620 (*p* = 1.29 × 10^−7^) on chromosome 1, which was annotated with the gene *CD46. CD46* gene encodes a type 1 membrane protein, which protects host cells from injury by complement and modulates T-cell activation [31].

* In the top panel, Manhattan plot shows the Cortical Epigenetic Clock GWAS results for ROSMAP. The bottom panel shows the GWAS results for the meta-analysis combining ROSMAP and BDR. Dashed red line represents a suggestive threshold of *p* = 10^−5^. The solid-line represents the genome-wide significance level of *p* = 5 × 10^−8^. Labels show the closest genes to GWAS lead SNPs with MAF >5%.

**Table 2.** Leading SNPs from GWAS of cortical clock: meta-analysis of ROSMAP and BDR.

	Chr:pos	Effect allele	Non-effect	EAF	beta	p-value	Gene annotation	
Rs4844620	1:207980901	A	G	0.12	0.886	1.29E-07	CD46	Membrane cofactor 9, protects injury by complement, modulates T cell activation. inflammatory disorders, cancers
rs17187636	2:166309615	A	G	0.08	1.266	1.17E-06	CSRNP3, GALNT3, FIGN	CSRNP3: DNA-binding transcription factor, apoptotic processes and transcriptional activity, related to education, bone density GALNT3: oligosaccharide biosynthesis, phosphate homeostasis FIGN: microtubule organization, locomotion, neuropathy
rs17743504	17:10080837	A	T	0.12	1.008	1.47E-06	GAS7	In purkinje neurons, intraocular pressure, glaucoma
Rs34403329	8:18029698	A	G	0.35	−0.826	2.48E-06	NAT1	Folate catabolism, bladder cancer, urate, depression, blood pressure
Rs13180000	5:50132506	A	T	0.22	−0.825	1.66E-06	PARP8	Protein ADP-ribosylase activity, diabetes, complement component C6
rs9557340	13:100580750	A	G	0.32	0.713	2.97E-06	CLYBL	Mitochondrial enzyme, regulation of cobalamin metabolic process, vitamin B12 levels
rs2593685	11:22391419	T	C	0.41	0.671	3.65E-06	SLC17A6	Neurotransmitter transporter, synaptic activity
rs4979892	10:79341734	T	C	0.46	−0.626	6.39E-06	KCNMA1 DLG5	KCNMA1: potassium channel, smooth muscle contraction, neurotransmitter release, neuron excitability, epilepsy DLG5: cell scaffolding, cell proliferation, dendritic spine formation, synaptogenesis
rs4253425	4:187205929	A	C	0.10	0.959	6.56E-06	F11 CYP4V2 KLKB1	F11: coagulation factor XI, clotting, VTE, TPA levels
rs836815	5:79995116	A	G	0.30	−0.679	7.52E-06	MSH3	DNA repair, cortical thickness, huntington disease, endometrial cancer
rs13105396	4:17047358	T	C	0.06	1.445	7.79E-06	CPB2	Hydrolyze c-terminal peptide bonds, downregulates fibrinolysis, complement cascade, thrombosis
rs79932664	21:43779709	T	C	0.09	1.038	7.87E-06	TFF1	Secretory protein
rs17111206	1:94646439	A	G	0.06	1.321	9.39E-06	ARHGAP29	Rho GTPase signaling, cleft palate

In secondary analyses of our GWAS of cortical clock age in ROSMAP specimens, with a larger number of SNPs than available for the meta-analysis, we identified 304 SNPs meeting our predefined criteria for suggestive statistical significance (*p* < 10^−5^) (Figure 2 and eTable 2). We found no evidence of genomic inflation (eFigure 1). After excluding SNPs with effect allele frequency less than 5%, we identified ‘leading’ SNPs from 24 independent loci. Table 3 shows the 24 lead SNPs with the highest statistical significance for each independent loci. The top SNP was rs4721030, which was close to genome-wide significant (*p* = 8.64 × 10^−8^). This SNP was annotated with two nearby genes, *TMEM106B* (transmembrane protein 106B) and *THSD7A* (thrombospondin type 1 domain containing 7A) on chromosome 7. *TMEM106B* gene appears to be a key regulator of aging, including influences on microglial proliferation and survival and involvement in myelination and lysosomal pathways [32]. *TMEM106B* has been previously associated with Alzheimer’s dementia and other neurodegenerative phenotypes in multiple studies (i.e., TDP-43 proteinopathy, brain inflammation, and amyloid deposition in the brain) [30,32–37]; further, our own previous research in ROSMAP has found links of *TMEM106B* with LATE-NC and worse cognitive resilience to neuropathology [16,38].

**Table 3.** Leading SNPs from GWAS of cortical clock: ROSMAP.

	Chr:pos	Effect allele	Non-effect	EAF	Beta	p-value	Gene annotation	
rs4721030	7:12137067	G	T	0.09	8.728	8.64E-08	THSD7A, TMEM106B	THSD7A: membrane-associated N-glycoprotein, neurovasculature, education TMEM106B: microglial survival/proliferation, myelination, lysosomal pathwyas, neurodegeneration
rs13180000	5:50132506	A	T	0.22	−0.993	8.44E-07	PARP8	See above
Rs3788369	22:24892973	G	A	0.05	2.863	1.36E-06	UPB1	Pyrimidine degradation pathway
Rs11998660	8:16546686	G	A	0.14	8.594	1.37E-06	FGF20	Fibroblast growth factor, mitogenesis, cell survival, neurotrophic factor, CNS development and function
Rs12386903	8:18000966	C	G	0.35	−1.770	1.47E-06	NAT1, ASAH1	ASAH1: acid ceramidase family, innate immune system, sphingolipid metabolism, cell signaling, seizures
Rs4943525	13:38206239	T	C	0.57	0.791	1.57E-06	TRPC4	Cation channels, endothelial permeability, vasodilation, neurotransmitter release, epilepsy
rs6925799	6:132467591	G	A	0.27	4.489	1.60E-06	MOXD1	Dopamine catabolic process, endoplasmic reticulum membrane protein,
rs7032313	9:36927081	T	C	0.52	−0.763	2.13E-06	PAX5	Transcription factor, B lymphocytes
rs73133134	12:76420006	T	C	0.12	−1.112	2.38E-06	PHLDA1	Apoptosis, neural development, IGF-1
rs10761557	10:62523410	G	A	0.29	0.800	2.49E-06	CDK1	Protein kinase complex, M phase promoting factor
rs4844620	1:207980901	A	G	0.12	0.916	2.86E-06	CD46	See above
rs1343559	13:87264407	G	C	0.88	−0.925	2.96E-06	SLITRK5	Membrane protein, homologous with neurotrophin receptors
rs77221864	7:152650529	G	A	0.06	1.508	3.09E-06	ACTR3B	Actin-related proteins, cytoskeleton, cell motility, neuropathy
rs34403329	8:18029698	A	G	0.15	−0.955	3.13E-06	NAT1	See above
rs7731137	5:154005654	G	T	0.57	−0.885	3.50E-06	LARP1	RNA binding protein, mTORC1 complex, growth signals, nutrient availability
rs11250535	10:1504987	G	C	0.21	−0.858	3.61E-06	ADARB2	RNA editing enzyme, diabetes, NFT, ALS
rs9315498	13:38099921	A	C	0.39	−0.736	4.27E-06	POSTN	Extracellular matrix protein, tissue development and regeneration, asthma
rs114109920	12:50159398	A	G	0.07	7.0979	4.51E-06	TMBIM6	Negative regulation of RNA metabolic processes and of intrinsic apoptotic signaling pathway
rs77378270	19:56229834	C	T	0.07	−1.296	5.46E-06	NLRP9	Innate immune system, inflammation
rs116430246	2:158855781	T	C	0.08	1.881	5.47E-06	UPP2	Cleavage of uridine and deoxyuridine to uracil and ribose
rs1596489	6:92915192	T	G	0.16	−0.951	5.95E-06	EPHA7	Protein tyrosine kinase family, axon guidance, brain development, cancers
rs6752755	2:175093901	G	C	0.15	0.882	7.42E-06	OLA1	GTPase protein family, Cancer (breast and ovarian)
rs55639005	8:74628306	C	T	0.12	−0.920	8.12E-06	STAU2	dsDNA binding protein, transport of neuronal RNA from cell body to dendrite
rs10812360	9:26190219	C	T	0.55	−0.737	8.56E-06	LOC100506422	

Most importantly, several SNPs were identified both in ROSMAP and in the meta-analysis of ROSMAP/BDR, suggesting consistent findings across the cohorts. In particular, rs4244620, was a leading SNP in both, including the strongest association in the meta-analysis. In addition, both sets of analyses pointed to rs34403329 on chromosome 8 (*p* = 2.48 × 10^−6^), which was annotated with two nearby genes, *NAT1* (N-acetyltransferase 1) and *ASAH1* (N-acylsphingosine amidohydrolase 1), and rs1318000 on chromosome 5 (*p* = 1.66 × 10^−6^), which was annotated to the gene *PARP8* (poly[ADP ribose] polymerase family member 8). (Note: The SNP with the strongest p-value from the GWAS of ROSMAP, rs4721030, was not in the BDR data and thus was not be tested in the meta-analysis.)

In sensitivity analyses, in models controlling for neuron proportion, we did not find any meaningful differences in the relations of leading SNPs to cortical clock age (eTable 3). Results were also largely consistent with analyses controlled for seven different cell-type proportions (eTable 4), which were estimated using single-cell methylation sequencing as the reference.

### Expression quantitative trait loci (eQTL)

**Figure 3.**
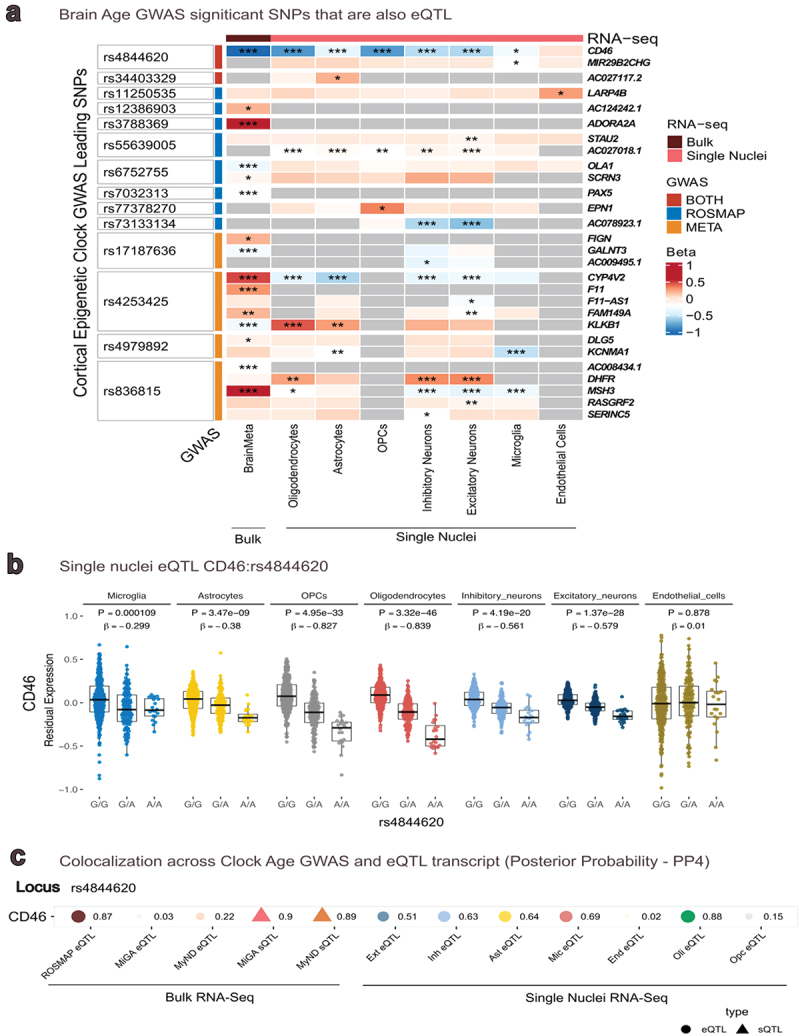
Expression quantitative trait Loci** in **panel a**, Heatmap show the GWAS lead SNPs that are FDR significant eQTL in bulk brain RNAseq (BrainMeta), and sn-RNAseq cell specific eQTL. *P ≤ 0.05, **P ≤ 0.01, ***P ≤ 0.001. The strongest relation of SNP to expression level was identified for rs4844620 with CD46. In **panel b**, we show CD46 expression levels across genotypes of rs4844620 in 7 cell types from ROSMAP single nucleus RNAseq. This was the strongest finding for the snRnaseq results. In **panel c**, colocalization analyses demonstrate the likelihood that a SNP is causal for both cortical clock age and for expression levels. The value to the right of each point is the posterior probability (PP) that the SNP is related to clock age and to the QTL; PP > 0.8 is standardly considered as high likelihood the SNP is causal for both traits. ROSMAP eQTL are from frontal cortex; MiGA are from primary monocytes; MyND are primary microglia; single cell eQTL are from ROSMAP frontal cortex.

Next, we leveraged expression QTLs derived from bulk RNA-Seq data in 2,865 cortex specimens from BrainMeta, to examine the leading SNPs in meta-analysis and in ROSMAP GWAS (Figure 3a). In the 13 lead SNPs from the ROSMAP/BDR meta-analysis, we found that five SNPs (rs4844620, rs17187637, rs4979892, rs4253425, rs836815) exhibited an FDR-significant cis-eQTL effect (Figure 3a) in 8 transcripts in the ROSMAP bulk transcriptomic data. From the leading SNPs in ROSMAP, we found that five SNPs (rs4844620, rs6752755, rs7032313, rs12386903, rs3788369) exhibited an FDR-significant cis-eQTL effect in five transcripts (*CD46, OLA1, PAX5, ASAH, ADORA2A*) (Figure 3a). Overall, transcripts included genes involved in a range of pathways, including inflammation and immune response (*CD46*, [31] *PAX5*, [39] *ADORA2A* [40], energetics (*OLA1* [41]), signal transduction (*DLG5* [42]), clotting (*F11* [43]) and DNA repair (*MSH3* [44]).

We also examined eQTLs in seven cell types from the single nucleus RNA-Seq in ROSMAP cortex (Figure 3b and eTable 5). Most interestingly, we found that rs4844620 exhibited significant eQTL effects for *CD46* gene expression in six cell types, such that each additional effect allele was associated with lower *CD46* expression (Figure 3b). The strongest associations were observed in oligodendrocyte precursor cells (*p* = 4.95 × 10^−33^) and in oligodendrocytes (*p* = 3.32 × 10^−46^). For the SNPs from the ROSMAP/BDR meta-analysis, there were no further significant single nucleus eQTLs beyond those for rs4844620.

### Colocalization across clock age and quantitative trait loci

To better evaluate whether lead SNPs may be associated with both clock age as well as gene expression, we examined colocalization for the lead SNPs and five different QTL datasets (Figure 3c), with a focus on immune systems: eQTL from our ROSMAP DLPFC and eQTL and sQTL from MyND (sorted monocytes) and MiGA (microglia). We found that rs4844620 (posterior probability > 0.8) colocalized across clock age, *CD46* eQTL in ROSMAP brain tissue as well as *CD46* sQTL in both monocytes and microglia. This suggests that rs4844620 is a common causal variant of clock age as well as the three different QTLs, providing support for a role of the *CD46* gene and immune pathways in DNA methylation aging in brain.

We also conducted colocalization analyses across our lead SNPs and the seven cell types in the ROSMAP single-nucleus eQTL (Figure 3c). We found that observed associations with cortical clock age and with *CD46* expression in oligodendrocytes appeared to be due to the single colocalizing variant rs4844620 (PP > 0.8). Thus, the *CD46* gene with respect to oligodendrocyte biology may have particular relevance.

### Functional analysis

When we used snpXplorer to examine biologic processes that may underlie cortical clock age, in the lead SNPs, there were no significant findings after correction for multiple comparisons. Nonetheless, two pathways were nominally significant (eTable 6): drug metabolism (*p* = 0.009) and pyrimidine nucleoside catabolic processes (*p* = 0.04).

With snpXplorer, we also identified SNPs and genes that overlapped with previous GWAS association studies. We found that rs4844620 was reported in previous GWAS of age-related macular degeneration. In addition, several genes from our GWAS were previously related to type 2 diabetes and to waist circumference, as well as to depression and cognitive function. These results may suggest that metabolism or metabolic dysregulation underlie DNA methylation age in brain.

### Aging phenotypes

**Figure 4.**
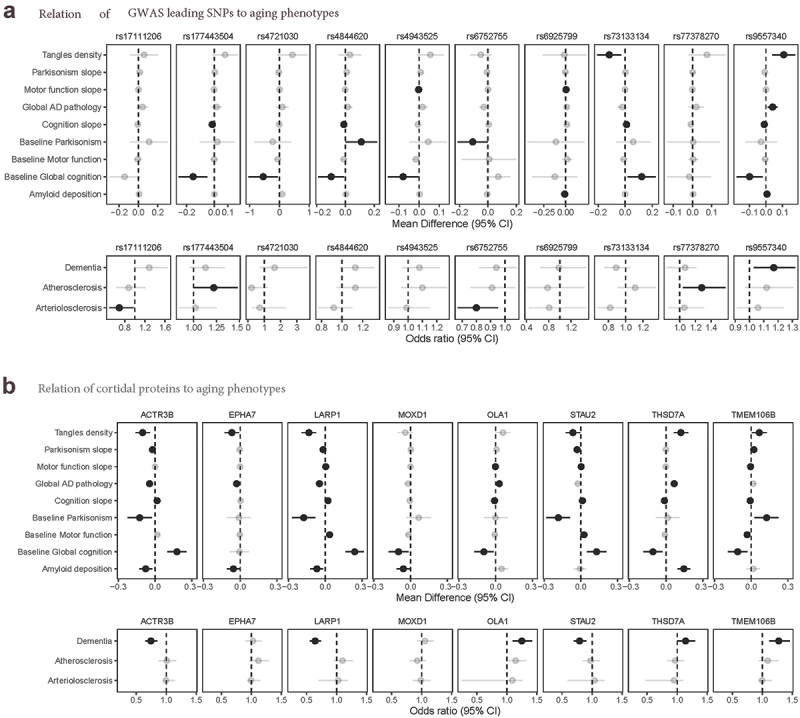
Relations of SNPs and cortical proteins to aging Phenotypes**, **panel a**: we show relations of leading SNPs to aging phenotypes, for SNPs with nominally significant relations with phenotypes. For continuous outcomes, we show mean differences from linear or linear mixed effects models; for categorical outcomes, we show odds ratios from logistic regression models. All models controlled for age at death, sex, and education. Of particular interest here, rs4844620 was related to lower baseline cognition (*p* = 0.031), steeper slopes of cognitive decline over time (*p* = 0.007), higher baseline levels of Parkinsonian signs (*p* = 0.046). Additionally, rs9557340 was related to more global AD neuropathology (*p* = 0.0047), more amyloid-β load (*p* = 0.014), greater PHFtau tangle density (*p* = 0.0022), lower baseline cognition (*p* = 0.014) and higher odds of dementia (*p* = 0.0038). **panel b**: we were able to examine protein levels in frontal cortex, based on annotated genes in our GWAS (14 genes of interest had corresponding protein levels in our proteomics data). We show here results for proteins which reached nominal significance for phenotypes. Of particular interest here, higher levels of the protein THSD7A in prefrontal cortex were related to more global AD pathology (*p* < 10^−5^), more amyloid-β load (*p* < 10^−5^), greater PHFtau tangle density (*p* < 10^−5^), lower baseline cognition (*p* = 0.007), steeper slopes of cognitive decline (*p* = 0.001) and higher odds of dementia (*p* = 0.046). Higher levels of TMEM106B protein were related to greater PHFtau tangle density (*p* = 0.03), more parkinsonism at baseline (*p* = 0.01) and steeper slopes over time (*p* = 0.0001), lower motor function at baseline (*p* = 0.001) and steeper slopes (*p* < 10^−5^), lower baseline cognition (*p* = 0.007), steeper slopes of cognitive decline (*p* = 0.04) and higher odds of dementia (*p* = 0.0004). Solid colors indicate significant association.

In our last steps, to help identify clinical relevance, we examined if each leading SNP was associated with aging phenotypes measured in the ROSMAP cohorts, after controlling for age at death, sex, education, and cohort (eTable 7). It is difficult to determine appropriate correction for multiple comparisons, since many of these aging outcomes are correlated with each other; we considered nominally significant findings for any phenotype (Figure 4a) and focus here on the strongest results. Perhaps most interesting, rs4844620 (which was identified in the ROSMAP GWAS, the ROSMAP/BDR meta-analysis, and exhibited cis-eQTL effects for *CD46* in the bulk RNA-Seq and snRNA-Seq, as described above), was associated with lower level of baseline cognition (β=-0.10, *p* = 0.030), faster slopes of cognitive decline (β=-0.01, *p* = 0.007), and with greater level of Parkinsonian signs at baseline (β = 0.11, *p* = 0.04), indicating pleiotropic relations with cognitive and motor phenotypes. Among additional leading SNPs, rs9557340 was related to multiple neuropathologies and to cognition. Specifically, this SNP was associated with higher global AD pathologic burden (β = 0.04, *p* = 0.0047), greater β-amyloid load (β = 0.007, *p* = 0.014), greater tau tangle density (β = 0.11, *p* = 0.0022), higher odds of dementia (odds ratio = 1.17, *p* = 0.016), and to faster slopes of cognitive decline (β=-0.10, *p* = 0.014).

Finally, using available proteomics data in over 800 ROSMAP participants, we were able to select 14 proteins measured in DLPFC (ACTR3B, ASAH1, CD46, EPHA7, LARP1, MOXD1, OLA1, NAT1, SLITRK5, POSTN, STAU2, TMEM106B, THSD7A, TMBIM6), which corresponded to annotated genes or eQTLs for lead SNPs in our GWAS. We examined the relation of these 14 proteins with neuropathologies, cognitive, and motor phenotypes, after controlling for age at death, sex, education, and cohort (eTable 8). Again, we considered any nominally significant relation of protein level to phenotypes, and focus here on the strongest findings (Figure 4B). Notably, our GWAS SNP rs4721030 on chromosome 7 was annotated with two genes, *TMEM106B* and *THSD7A*; we found broad relations of both proteins to most of the aging phenotypes we examined. In particular, TMEM106B protein levels were strongly related to odds of dementia (*p* = 0.0004) and lower level of cognitive function at baseline (*p* = 0.007), as well as to worse motor function (motor function at baseline, *p* = 0.001; slopes of decline, *p* < 10^−5^) and more Parkinsonian signs (baseline, *p* = 0.01; slopes, *p* < 10^−5^). THSD7A protein levels were strongly to greater global AD pathology, to greater amyloid-β load and PHFtau tangle density (all *p* < 10^−5^), as well as to lower level of cognitive function at baseline (*p* = 0.007) and faster slopes of cognitive decline (*p* = 0.001).

## Discussion

We conducted a GWAS of cortical epigenetic clock age calculated in brain tissue, among 1,340 older, deceased participants from three cohorts; we further leveraged transcriptomic, proteomic, and phenotypic data from participants, as well as publicly available data, to identify converging evidence of genes underlying cortical epigenetic clock age. In particular, our results supported several genes, especially *CD46*, as candidates of interest with potentially broad roles in cortical clock age.

Several previous studies have also conducted GWAS of epigenetic clocks in both peripheral blood and brain tissue [45–48]. Consistent with our findings, the largest GWAS of five clocks, calculated in blood samples (*n* = 40,000),^45^ prioritized *CD46* as a possible determinant of epigenetic age from the Horvath clock. Additionally, *CLYBL* (Citramalyl-CoA Lyase) was prioritized as a predictor of the PhenoAge clock; we found that a loci (rs9557340) in the *CLYBL* gene was related to cortical clock age and was strongly related to AD neuropathologies and to cognition – suggesting clinical relevance of *CLYBL* gene to brain aging. However, in a GWAS (*n* = 1100) of the Horvath clock in brain tissue (which included some of our ROSMAP participants) [47], there was no overlap of their primary findings in prefrontal cortex with ours for the cortical clock; these samples spanned a much wider age range than ours, and there may be differences in genetic correlates of aging at different ages. Nonetheless, overall, there is some evidence that specific genes may have systemic effects on biologic aging.

In terms of our own findings, *CD46* gene encodes the membrane cofactor protein, an ubiquitously expressed complement regulatory protein. *CD46* protects host cells from injury by complement and appears to link innate and adaptive immune responses. The complement system regulates central nervous system function, and complement-mediated neuroinflammation may be involved in a range of aging-related neurodegenerative disorders, from cognitive impairment to age-related macular degeneration [49]. Other genes in the complement system are strongly related to dementia, including *CR1, C1S, CD33,* and *TREM2*; interestingly, *CR1*, a well-established genetic risk factor for dementia, is a receptor for complement C3b and C4b proteins, and *CD46* mediates inactivation of C3b and C4b, pointing to potential mechanistic pathways [50]. Further, in our results, rs4844620 appeared to be a common causal SNP across epigenetic age and eQTLs for *CD46* in cortex tissue, as well as in primary monocytes and microglial samples, supporting the role of *CD46* in immune response in the brain. In addition, our single nucleus data from DLPFC further identified eQTL effects for *CD46* in oligodendrocytes, and colocalization of this SNP with epigenetic age and CD46 expression in oligodendrocytes. This may support recent data that oligodendrocytes could have immunomodulatory properties [51]. Overall, there is compelling evidence, from our study and others, to support *CD46* as a target for further research.

In additional results from the GWAS of ROSMAP alone, the strongest loci in relation to clock age in our GWAS was rs4721030 on chromosome 7, which was associated with older epigenetic age, and nearly reached genome-wide significance. We note that the beta value we found for this SNP in relation to clock age was 8.7, which is much larger than many of our other findings, indicating more caution is warranted in interpreting this result. For context, the mean beta value in our 304 SNPs of interest in ROSMAP was 2.6 (SD 2.5); that is, the beta for rs4721030 was 2.4 standard deviations greater than the mean beta in our research here, which is large although not an extreme outlier. Further, we could not examine this SNP in the meta-analysis, since it was not present in the BDR data, even after imputation, likely due to the highly specialized array used by BDR. For these reasons, this finding should be carefully considered and interpreted cautiously. Nonetheless, the SNP was annotated with two genes, *TMEM106B* and *THSD7A*. Previous work has implicated the *TMEM106B* gene in neurodegenerative phenotypes, especially TDP-43 pathology [38]. *THSD7A* is a soluble form of membrane-associated N-glycoprotein, produced by cells of endothelial and neuronal origin. In zebrafish, *THSD7A* may have a role in the neurovasculature [52], and *THSD7A* has been related to educational attainment [53]; in recent research using GWAS summary statistics, *TMEM106B/THSD7A* were reported as pleiotropically related to dementia and major depression disorder [54]. Interestingly, we found associations of higher THSD7A/TMEM106B protein levels in ROSMAP DLPFC with a wide array of aging phenotypes, from AD pathologies to lower cognitive function to worse motor function. Thus, our findings in GWAS as well as in cortical protein levels, together with the existing literature, may suggest that *TMEM106B and THSD7A* have relevance to brain aging.

There are substantial strengths to our work here. We assembled a sample of older individuals for this GWAS of cortical clock age; the uniform older ages of participants may be particularly important for evaluating the genetic architecture of cortical clock age, as there is substantial evidence that epigenetic clocks function differently at older than at younger ages [3,6]. We also had available a wide array of genomic and phenotypic data; this enabled us to validate and prioritize genes of interest despite the absence of a replication dataset. Limitations of our study should also be considered. Most importantly, our sample size was small for detecting genome-wide significant findings. However, many SNPs achieved a predetermined, ‘suggestive’ threshold of 10^−5^, which we [16] and others [55] have previously used. Further, to prioritize candidate loci, we were able to validate results with a range of additional information, from gene expression (both bulk and single-cell expression) to protein levels in brain tissue and evaluation of neuropathologic and clinical aging phenotypes. Thus, we were able to confirm findings via multiple lines of converging evidence. Nonetheless, we likely missed important SNPs which did not meet even our suggestive threshold. Second, the DNAm states as well as the transcriptomic and proteomic data were all derived from cortex. Thus, we may have missed genes that are primarily relevant in other brain regions. However, we focus on DLPFC given its critical role in a wide range of higher order human behaviour; additionally, evidence suggests a good correlation of epigenetic age across brain regions [4,47]. Finally, another limitation is our primarily non-Latino white participants, across all three cohorts. Evidence indicates that neurodegenerative diseases of aging, such as dementia, differ across diverse groups; for example, older African Americans appear to have higher risk of dementia, and Latinx individuals appear to develop dementia at younger ages, than non-Hispanic whites. [56,57]. Thus, future research is clearly needed in minoritized older participants to better understand brain aging in groups who often have worse health.

## Supplementary Material

-) tables supplementray 0724.docx

figure_suppl.docx
