# Supplementary material for: Genetic architecture of epigenetic cortical clock age in brain tissue from older individuals: alterations in CD46 and other loci
Source: Epigenetics. 2024 Aug 22;19(1):2392050. doi: 10.1080/15592294.2024.2392050 (PMC11346548; doi:10.1080/15592294.2024.2392050)
Supplement: -) tables supplementray 0724.docx [file KEPI_A_2392050_SM6885.docx]

SUPPLEMENTARY TABLES

eTable 1. Cortical Clock GWAS, 110 SNPs meeting suggestive significance (p<10^-5^) in the ROSMAP/BDR dorsolateral prefrontal cortex (n=1216)

eTable 2. Cortical Clock GWAS, 309 SNPs meeting suggestive significance (p<10^-5^) in the ROSMAP dorsolateral prefrontal cortex (n=694)

eTable 3. Leading SNPs from Cortical Clock GWAS in ROSMAP, controlling for neuron proportion

eTable 4. Leading SNPs from Cortical Clock GWAS in ROSMAP, controlling for seven cell type proportions

eTable 5. Expression quantitative trait loci from single nucleus RNA-seq in dorsolateral prefrontal cortex (n=424)

eTable 6. Enrichment for biologic processes in GO and KEGG

eTable 7. Relation of Leading SNPs to Aging Phenotypes (for SNPs which were related to an aging phenotype, at nominal significance)

eTable 8. Relation of Cortical Protein Levels to Aging Phenotypes (for proteins measured in cortex, and for proteins which were related to an aging phenotype, at nominal significance)

eTable 1. **Cortical Clock GWAS: SNPs meeting suggestive significance (p<10^-5^), ROSMAP/BDR Meta-analysis**

| Chr | Pos | EA | non_EA | EAF | Beta | SE | P-value | Callrate | Imputed | Oevar_imp | rsid |
| --- | --- | --- | --- | --- | --- | --- | --- | --- | --- | --- | --- |
| 1 | 207980901 | A | G | 0.12 | 0.8864 | 0.17 | 1.29E-07 | 0.995 | 1 | 0.966 | rs4844620 |
| 1 | 207983745 | A | C | 0.82 | -0.8452 | 0.17 | 3.65E-07 | 0.998 | 1 | 0.990 | rs66532523 |
| 1 | 207979577 | A | T | 0.18 | 0.8451 | 0.17 | 3.67E-07 | 0.999 | 1 | 0.993 | rs11118612 |
| 1 | 207975949 | T | C | 0.82 | -0.8357 | 0.17 | 5.03E-07 | 0.999 | 1 | 0.992 | rs56075814 |
| 1 | 208028029 | T | C | 0.79 | -0.8139 | 0.16 | 6.02E-07 | 0.997 | 1 | 0.988 | rs11586197 |
| 1 | 207934849 | A | G | 0.88 | -0.8269 | 0.17 | 6.31E-07 | 0.997 | 1 | 0.976 | rs4844390 |
| 1 | 207959070 | T | C | 0.89 | -0.8279 | 0.17 | 6.34E-07 | 0.996 | 1 | 0.972 | rs11118580 |
| 1 | 207991209 | C | G | 0.82 | -0.8272 | 0.17 | 6.60E-07 | 0.998 | 1 | 0.988 | rs4844392 |
| 1 | 208035434 | A | T | 0.20 | 0.8126 | 0.16 | 6.70E-07 | 0.992 | 1 | 0.966 | rs4844623 |
| 1 | 208035446 | A | G | 0.80 | -0.8125 | 0.16 | 6.71E-07 | 0.992 | 1 | 0.966 | rs4844624 |
| 1 | 208037821 | T | C | 0.21 | 0.8122 | 0.16 | 6.76E-07 | 0.995 | 1 | 0.977 | rs12145290 |
| 1 | 207960708 | T | C | 0.19 | 0.8239 | 0.17 | 6.90E-07 | 0.998 | 1 | 0.991 | rs4844619 |
| 1 | 207920788 | T | C | 0.81 | -0.8199 | 0.17 | 6.92E-07 | 0.998 | 1 | 0.991 | rs2796265 |
| 1 | 207923081 | A | G | 0.19 | 0.8201 | 0.17 | 6.92E-07 | 0.997 | 1 | 0.985 | rs2761437 |
| 1 | 207960104 | T | G | 0.19 | 0.8223 | 0.17 | 7.24E-07 | 0.999 | 1 | 0.992 | rs6657476 |
| 1 | 207934487 | A | C | 0.20 | 0.8183 | 0.17 | 7.87E-07 | 0.999 | 1 | 0.993 | rs2466572 |
| 1 | 207930203 | A | G | 0.82 | -0.8177 | 0.17 | 7.88E-07 | 1.000 | 1 | 0.999 | rs2724384 |
| 1 | 207943158 | T | C | 0.80 | -0.8187 | 0.17 | 7.89E-07 | 0.998 | 1 | 0.989 | rs2724360 |
| 1 | 207941191 | T | G | 0.80 | -0.8184 | 0.17 | 7.96E-07 | 0.998 | 1 | 0.990 | rs2724374 |
| 1 | 207917499 | A | G | 0.19 | 0.8125 | 0.17 | 8.87E-07 | 0.997 | 1 | 0.983 | rs2761434 |
| 1 | 208030303 | A | G | 0.83 | -0.8003 | 0.16 | 9.21E-07 | 0.999 | 1 | 0.993 | rs4844621 |
| 1 | 208029947 | T | C | 0.16 | 0.8003 | 0.16 | 9.27E-07 | 1.000 | 1 | 1.000 | rs7550821 |
| 1 | 208027696 | T | C | 0.17 | 0.7995 | 0.16 | 9.71E-07 | 0.998 | 1 | 0.988 | rs61821315 |
| 1 | 208033706 | T | C | 0.16 | 0.7980 | 0.16 | 1.08E-06 | 0.997 | 1 | 0.985 | rs61821318 |
| 1 | 208036509 | T | C | 0.16 | 0.7980 | 0.16 | 1.08E-06 | 0.996 | 1 | 0.979 | rs882198 |
| 1 | 208034329 | T | C | 0.16 | 0.7968 | 0.16 | 1.13E-06 | 0.997 | 1 | 0.982 | rs4844622 |
| 2 | 166309615 | A | G | 0.04 | 1.2656 | 0.26 | 1.17E-06 | 0.996 | 1 | 0.926 | rs17187636 |
| 1 | 208030856 | T | C | 0.16 | 0.7952 | 0.16 | 1.18E-06 | 0.999 | 1 | 0.994 | rs7551724 |
| 1 | 208031234 | A | G | 0.16 | 0.7952 | 0.16 | 1.18E-06 | 0.998 | 1 | 0.990 | rs12141901 |
| 1 | 208037967 | A | G | 0.10 | 0.8230 | 0.17 | 1.21E-06 | 0.997 | 1 | 0.975 | rs4844395 |
| 2 | 166312556 | T | C | 0.96 | -1.2635 | 0.26 | 1.28E-06 | 0.997 | 1 | 0.932 | rs2048740 |
| 1 | 208039471 | C | G | 0.16 | 0.7926 | 0.16 | 1.32E-06 | 0.993 | 1 | 0.965 | rs1967689 |
| 1 | 208007277 | T | G | 0.82 | -0.8101 | 0.17 | 1.41E-06 | 0.999 | 1 | 0.994 | rs61821293 |
| 17 | 10080837 | A | T | 0.91 | 1.0083 | 0.21 | 1.47E-06 | 0.987 | 1 | 0.880 | rs17743504 |
| 2 | 166337811 | T | C | 0.04 | 1.2568 | 0.26 | 1.65E-06 | 0.996 | 1 | 0.920 | rs79220360 |
| 5 | 50132506 | A | T | 0.22 | -0.8253 | 0.17 | 1.66E-06 | 0.986 | 1 | 0.933 | rs13180000 |
| 5 | 50144735 | A | C | 0.22 | -0.8469 | 0.18 | 2.07E-06 | 0.983 | 1 | 0.925 | rs1862547 |
| 2 | 166385362 | A | C | 0.96 | -1.2793 | 0.27 | 2.22E-06 | 0.993 | 1 | 0.876 | rs78659826 |
| 5 | 50149333 | A | G | 0.21 | -0.8440 | 0.18 | 2.29E-06 | 0.984 | 1 | 0.929 | rs62365519 |
| 5 | 50152455 | A | G | 0.22 | -0.8417 | 0.18 | 2.40E-06 | 0.982 | 1 | 0.921 | rs16886818 |
| 8 | 18029698 | A | G | 0.15 | -0.8263 | 0.18 | 2.48E-06 | 0.996 | 1 | 0.975 | rs34403329 |
| 1 | 208014922 | T | C | 0.82 | -0.7883 | 0.17 | 2.51E-06 | 1.000 | 1 | 0.999 | rs1318653 |
| 1 | 208017915 | T | C | 0.18 | 0.7872 | 0.17 | 2.61E-06 | 0.998 | 1 | 0.988 | rs11118668 |
| 1 | 208018779 | A | T | 0.18 | 0.7867 | 0.17 | 2.65E-06 | 0.997 | 1 | 0.986 | rs55935450 |
| 2 | 166460229 | A | C | 0.95 | -1.2601 | 0.27 | 2.75E-06 | 0.994 | 1 | 0.895 | rs17251665 |
| 13 | 100580750 | A | G | 0.29 | 0.7134 | 0.15 | 2.97E-06 | 0.963 | 1 | 0.872 | rs9557340 |
| 2 | 166239891 | A | G | 0.02 | 1.6057 | 0.34 | 3.16E-06 | 0.997 | 1 | 0.886 | rs79891251 |
| 1 | 208025926 | T | C | 0.82 | -0.7809 | 0.17 | 3.18E-06 | 0.995 | 1 | 0.977 | rs6669384 |
| 5 | 50147620 | A | G | 0.21 | -0.8282 | 0.18 | 3.34E-06 | 0.985 | 1 | 0.931 | rs12659764 |
| 11 | 22391419 | T | C | 0.75 | 0.6705 | 0.14 | 3.65E-06 | 0.981 | 1 | 0.924 | rs2593685 |
| 5 | 50085743 | A | G | 0.79 | 0.8247 | 0.18 | 3.84E-06 | 0.999 | 1 | 0.994 | rs16885842 |
| 11 | 22402276 | A | G | 0.31 | -0.6621 | 0.14 | 3.84E-06 | 0.994 | 1 | 0.979 | rs2665699 |
| 11 | 22397739 | A | C | 0.31 | -0.6635 | 0.14 | 4.02E-06 | 0.993 | 1 | 0.974 | rs2248436 |
| 1 | 207994429 | T | C | 0.82 | -0.7584 | 0.17 | 4.27E-06 | 0.997 | 1 | 0.983 | rs4844393 |
| 5 | 50072860 | T | C | 0.79 | 0.8195 | 0.18 | 4.37E-06 | 0.999 | 1 | 0.993 | rs67151831 |
| 1 | 208026739 | T | G | 0.17 | 0.7421 | 0.16 | 4.41E-06 | 0.987 | 1 | 0.934 | rs7532674 |
| 5 | 50114421 | T | C | 0.21 | -0.8180 | 0.18 | 4.60E-06 | 0.992 | 1 | 0.961 | rs1125916 |
| 5 | 50127289 | A | T | 0.79 | 0.8178 | 0.18 | 4.62E-06 | 0.989 | 1 | 0.945 | rs13186089 |
| 11 | 22418570 | A | G | 0.31 | -0.6506 | 0.14 | 5.26E-06 | 0.988 | 1 | 0.962 | rs726859 |
| 11 | 22420611 | A | G | 0.69 | 0.6506 | 0.14 | 5.26E-06 | 0.990 | 1 | 0.966 | rs2593651 |
| 11 | 22421015 | T | C | 0.31 | -0.6506 | 0.14 | 5.27E-06 | 0.989 | 1 | 0.964 | rs2593652 |
| 5 | 50062611 | T | C | 0.21 | -0.8140 | 0.18 | 5.32E-06 | 0.999 | 1 | 0.994 | rs10512906 |
| 5 | 50065333 | T | C | 0.21 | -0.8137 | 0.18 | 5.39E-06 | 0.999 | 1 | 0.993 | rs34428476 |
| 5 | 50087466 | T | G | 0.21 | -0.8134 | 0.18 | 5.47E-06 | 0.999 | 1 | 0.994 | rs12654399 |
| 5 | 50066049 | C | G | 0.21 | -0.8130 | 0.18 | 5.47E-06 | 0.999 | 1 | 0.993 | rs1993955 |
| 5 | 50088850 | A | G | 0.21 | -0.8130 | 0.18 | 5.51E-06 | 0.999 | 1 | 0.995 | rs3806887 |
| 11 | 22411969 | C | G | 0.31 | -0.6494 | 0.14 | 5.64E-06 | 0.988 | 1 | 0.960 | rs721841 |
| 11 | 22411865 | T | C | 0.69 | 0.6493 | 0.14 | 5.66E-06 | 0.988 | 1 | 0.959 | rs721840 |
| 5 | 50051320 | A | G | 0.21 | -0.8132 | 0.18 | 5.72E-06 | 0.999 | 1 | 0.994 | rs113240270 |
| 5 | 50048822 | A | C | 0.21 | -0.8132 | 0.18 | 5.75E-06 | 0.999 | 1 | 0.993 | rs35328303 |
| 11 | 22418220 | T | G | 0.29 | -0.6486 | 0.14 | 5.75E-06 | 0.992 | 1 | 0.972 | rs726858 |
| 11 | 22422503 | C | G | 0.70 | 0.6486 | 0.14 | 5.75E-06 | 0.992 | 1 | 0.970 | rs2593653 |
| 11 | 22423166 | T | C | 0.29 | -0.6486 | 0.14 | 5.76E-06 | 0.993 | 1 | 0.974 | rs2593657 |
| 5 | 50071254 | T | C | 0.21 | -0.8109 | 0.18 | 5.81E-06 | 0.998 | 1 | 0.991 | rs62365481 |
| 11 | 22428882 | A | G | 0.71 | 0.6498 | 0.14 | 5.86E-06 | 0.995 | 1 | 0.980 | rs2665718 |
| 11 | 22417107 | A | G | 0.29 | -0.6482 | 0.14 | 5.92E-06 | 0.992 | 1 | 0.971 | rs2665713 |
| 2 | 166383675 | T | C | 0.95 | -1.1922 | 0.26 | 5.93E-06 | 0.993 | 1 | 0.879 | rs77939846 |
| 11 | 22408803 | T | C | 0.29 | -0.6502 | 0.14 | 6.05E-06 | 0.992 | 1 | 0.972 | rs2665705 |
| 11 | 22414861 | C | G | 0.29 | -0.6479 | 0.14 | 6.06E-06 | 0.992 | 1 | 0.971 | rs2665712 |
| 5 | 50043652 | A | G | 0.21 | -0.8118 | 0.18 | 6.10E-06 | 0.999 | 1 | 0.993 | rs62368285 |
| 11 | 22428384 | T | C | 0.71 | 0.6467 | 0.14 | 6.38E-06 | 0.995 | 1 | 0.980 | rs2593663 |
| 10 | 79341734 | T | C | 0.59 | -0.6256 | 0.14 | 6.39E-06 | 0.980 | 1 | 0.934 | rs4979892 |
| 5 | 50035910 | A | C | 0.21 | -0.8103 | 0.18 | 6.53E-06 | 0.999 | 1 | 0.993 | rs34241669 |
| 4 | 187205929 | T | C | 0.07 | 0.9591 | 0.21 | 6.56E-06 | 1.000 | 1 | 0.999 | rs4253425 |
| 5 | 50160386 | A | C | 0.41 | 0.7424 | 0.16 | 6.73E-06 | 0.974 | 1 | 0.918 | rs152001 |
| 11 | 22417712 | A | T | 0.33 | -0.6415 | 0.14 | 7.13E-06 | 0.987 | 1 | 0.958 | rs1447504 |
| 5 | 50024757 | A | C | 0.79 | 0.8072 | 0.18 | 7.36E-06 | 0.998 | 1 | 0.992 | rs13162945 |
| 5 | 79995116 | A | G | 0.24 | -0.6785 | 0.15 | 7.52E-06 | 0.994 | 1 | 0.975 | rs836815 |
| 5 | 80000152 | A | G | 0.25 | -0.6785 | 0.15 | 7.52E-06 | 0.994 | 1 | 0.974 | rs865644 |
| 5 | 50021795 | A | G | 0.21 | -0.8064 | 0.18 | 7.58E-06 | 0.998 | 1 | 0.991 | rs35571312 |
| 11 | 22435111 | A | C | 0.78 | 0.6477 | 0.14 | 7.61E-06 | 1.000 | 1 | 1.000 | rs3106185 |
| 5 | 49967825 | A | G | 0.22 | -0.8072 | 0.18 | 7.77E-06 | 0.998 | 1 | 0.989 | rs13166556 |
| 11 | 22424782 | A | C | 0.68 | 0.6393 | 0.14 | 7.78E-06 | 0.990 | 1 | 0.964 | rs2593660 |
| 4 | 17047358 | T | C | 0.97 | 1.4445 | 0.32 | 7.79E-06 | 0.996 | 1 | 0.912 | rs13105396 |
| 21 | 43779709 | T | C | 0.05 | 1.0375 | 0.23 | 7.86E-06 | 0.995 | 1 | 0.931 | rs79932664 |
| 5 | 49970496 | T | G | 0.79 | 0.8090 | 0.18 | 7.92E-06 | 0.998 | 1 | 0.990 | rs12653772 |
| 21 | 43780172 | T | C | 0.05 | 1.0394 | 0.23 | 7.93E-06 | 0.995 | 1 | 0.926 | rs111959595 |
| 5 | 50103818 | A | G | 0.21 | -0.7952 | 0.18 | 7.99E-06 | 0.995 | 1 | 0.975 | rs16877029 |
| 21 | 43780752 | A | G | 0.95 | -1.0418 | 0.23 | 7.99E-06 | 0.994 | 1 | 0.918 | rs13047691 |
| 5 | 79995948 | C | G | 0.76 | 0.6766 | 0.15 | 8.05E-06 | 0.994 | 1 | 0.975 | rs836814 |
| 11 | 22408655 | A | G | 0.67 | 0.6375 | 0.14 | 8.06E-06 | 0.986 | 1 | 0.957 | rs2665704 |
| 11 | 22405010 | T | G | 0.32 | -0.6379 | 0.14 | 8.25E-06 | 0.988 | 1 | 0.962 | rs2665701 |
| 5 | 49985525 | T | C | 0.21 | -0.8045 | 0.18 | 8.66E-06 | 0.997 | 1 | 0.987 | rs62366945 |
| 5 | 49988990 | A | T | 0.21 | -0.8038 | 0.18 | 8.76E-06 | 0.997 | 1 | 0.987 | rs61479216 |
| 5 | 49952219 | C | G | 0.22 | -0.8002 | 0.18 | 8.76E-06 | 0.997 | 1 | 0.985 | rs12654329 |
| 11 | 22406588 | C | G | 0.68 | 0.6342 | 0.14 | 8.86E-06 | 0.988 | 1 | 0.960 | rs2665702 |
| 4 | 187204937 | A | G | 0.07 | 0.9301 | 0.21 | 9.04E-06 | 0.999 | 1 | 0.992 | rs4253421 |
| 1 | 94646439 | A | G | 0.11 | 1.3208 | 0.30 | 9.39E-06 | 0.994 | 1 | 0.955 | rs17111206 |
| 5 | 50007911 | A | C | 0.79 | 0.7995 | 0.18 | 9.40E-06 | 0.998 | 1 | 0.988 | rs34389644 |
| 5 | 80029263 | A | G | 0.23 | -0.6729 | 0.15 | 9.61E-06 | 0.996 | 1 | 0.981 | rs368406 |

eTable 2. Cortical Clock GWAS, 304 SNPs meeting suggestive significance (p<10^-5^) in the ROSMAP dorsolateral prefrontal cortex (n=694)

| **Chr** | **Pos** | **EA** | **Non_EA** | **EAF** | **Beta** | **SE** | **Pvalue** | **Callrate** | **Imputed** | **Oevar_imp** | **rsid** |
| --- | --- | --- | --- | --- | --- | --- | --- | --- | --- | --- | --- |
| 7 | 12137067 | G | T | 0.09 | 8.7277 | 1.6304 | 8.64E-08 | 0.991 | 1 | 0.917 | rs4721030 |
| 9 | 112433705 | G | T | 0.02 | 2.3125 | 0.4398 | 1.46E-07 | 1.000 | 1 | 0.995 | rs117914628 |
| 10 | 11280775 | G | A | 0.01 | 8.2081 | 1.6206 | 4.08E-07 | 0.997 | 1 | 0.841 | rs7083181 |
| 7 | 12140516 | G | A | 0.01 | 9.2704 | 1.8493 | 5.36E-07 | 0.999 | 1 | 0.963 | rs74959441 |
| 7 | 12140976 | A | T | 0.01 | 9.0576 | 1.8227 | 6.72E-07 | 0.999 | 1 | 0.965 | rs76029431 |
| 7 | 12168608 | A | T | 0.13 | 8.5907 | 1.7430 | 8.28E-07 | 0.986 | 1 | 0.911 | rs111914437 |
| 5 | 50132506 | A | T | 0.22 | -0.9932 | 0.2017 | 8.44E-07 | 0.986 | 1 | 0.933 | rs13180000 |
| 7 | 12142167 | T | C | 0.02 | 8.6606 | 1.7732 | 1.04E-06 | 0.999 | 1 | 0.962 | rs79192861 |
| 7 | 12157051 | A | G | 0.06 | 8.5593 | 1.7541 | 1.06E-06 | 0.991 | 1 | 0.886 | rs17165595 |
| 7 | 12144938 | G | T | 0.06 | 8.5879 | 1.7600 | 1.06E-06 | 0.994 | 1 | 0.908 | rs73287166 |
| 7 | 12167060 | A | G | 0.06 | 8.5091 | 1.7478 | 1.12E-06 | 0.992 | 1 | 0.897 | rs4721037 |
| 7 | 12152927 | G | A | 0.06 | 8.5220 | 1.7570 | 1.23E-06 | 0.993 | 1 | 0.897 | rs73289344 |
| 7 | 12151476 | A | G | 0.01 | 8.5270 | 1.7581 | 1.23E-06 | 0.999 | 1 | 0.941 | rs17165580 |
| 7 | 12164993 | T | C | 0.06 | 8.4840 | 1.7494 | 1.24E-06 | 0.992 | 1 | 0.895 | rs4719293 |
| 7 | 12180081 | G | C | 0.02 | 8.4400 | 1.7443 | 1.31E-06 | 0.997 | 1 | 0.903 | rs78136575 |
| 7 | 12183221 | A | C | 0.02 | 8.4376 | 1.7443 | 1.32E-06 | 0.997 | 1 | 0.906 | rs147583365 |
| 7 | 12192588 | G | T | 0.01 | 8.4302 | 1.7443 | 1.35E-06 | 0.999 | 1 | 0.953 | rs17165661 |
| 7 | 12196588 | T | G | 0.02 | 8.4292 | 1.7443 | 1.35E-06 | 0.999 | 1 | 0.963 | rs17842078 |
| 7 | 12198191 | C | G | 0.02 | 8.4292 | 1.7443 | 1.35E-06 | 0.999 | 1 | 0.962 | rs80350965 |
| 7 | 12199771 | A | G | 0.02 | 8.4292 | 1.7443 | 1.35E-06 | 0.999 | 1 | 0.956 | rs76543628 |
| 7 | 12204600 | T | C | 0.02 | 8.4262 | 1.7444 | 1.36E-06 | 0.999 | 1 | 0.953 | rs116144350 |
| 22 | 24892973 | G | A | 0.05 | 2.8630 | 0.5927 | 1.36E-06 | 0.998 | 1 | 0.964 | rs3788369 |
| 8 | 16546686 | G | A | 0.14 | 8.5937 | 1.7796 | 1.37E-06 | 0.989 | 1 | 0.935 | rs11998660 |
| 8 | 16548014 | A | T | 0.14 | 8.5824 | 1.7781 | 1.39E-06 | 0.989 | 1 | 0.935 | rs7838882 |
| 22 | 24893676 | T | C | 0.05 | 2.8632 | 0.5933 | 1.40E-06 | 0.998 | 1 | 0.966 | rs5751890 |
| 22 | 24903283 | A | G | 0.01 | 2.9492 | 0.6112 | 1.40E-06 | 0.998 | 1 | 0.899 | rs139225143 |
| 22 | 24893336 | G | A | 0.05 | 2.8610 | 0.5932 | 1.41E-06 | 0.998 | 1 | 0.968 | rs3788371 |
| 8 | 16549200 | C | T | 0.13 | 8.5782 | 1.7786 | 1.41E-06 | 0.990 | 1 | 0.936 | rs17123488 |
| 22 | 24895952 | T | C | 0.01 | 2.9478 | 0.6118 | 1.45E-06 | 0.999 | 1 | 0.895 | rs2232864 |
| 8 | 18000966 | C | G | 0.35 | -1.1770 | 0.2444 | 1.47E-06 | 0.989 | 1 | 0.964 | rs12386903 |
| 13 | 38206239 | T | C | 0.57 | 0.7910 | 0.1647 | 1.57E-06 | 0.983 | 1 | 0.950 | rs4943525 |
| 8 | 16544575 | T | C | 0.15 | 8.5818 | 1.7881 | 1.59E-06 | 0.987 | 1 | 0.925 | rs73541879 |
| 6 | 132467591 | G | A | 0.27 | 4.4885 | 0.9355 | 1.60E-06 | 0.979 | 1 | 0.920 | rs6925799 |
| 22 | 24895416 | C | T | 0.05 | 2.8496 | 0.5940 | 1.61E-06 | 0.999 | 1 | 0.982 | rs5760457 |
| 8 | 16544478 | A | G | 0.15 | 8.5914 | 1.7932 | 1.66E-06 | 0.987 | 1 | 0.925 | rs61744845 |
| 6 | 132468518 | G | A | 0.26 | 4.4870 | 0.9370 | 1.68E-06 | 0.977 | 1 | 0.914 | rs9483389 |
| 6 | 132468648 | A | C | 0.26 | 4.4868 | 0.9372 | 1.69E-06 | 0.977 | 1 | 0.913 | rs9493189 |
| 22 | 24896014 | G | A | 0.05 | 2.8465 | 0.5948 | 1.70E-06 | 0.999 | 1 | 0.976 | rs2232865 |
| 8 | 16544477 | C | G | 0.13 | 8.6104 | 1.7994 | 1.71E-06 | 0.990 | 1 | 0.937 | rs61744846 |
| 6 | 132469029 | C | T | 0.26 | 4.4870 | 0.9380 | 1.72E-06 | 0.977 | 1 | 0.913 | rs9493191 |
| 5 | 50085743 | G | A | 0.21 | -1.0032 | 0.2099 | 1.75E-06 | 0.999 | 1 | 0.994 | rs16885842 |
| 6 | 132469378 | C | T | 0.26 | 4.4861 | 0.9390 | 1.77E-06 | 0.977 | 1 | 0.912 | rs9493192 |
| 2 | 6554222 | A | G | 0.01 | 3.0112 | 0.6303 | 1.78E-06 | 0.995 | 1 | 0.659 | rs62110016 |
| 6 | 132469477 | A | G | 0.26 | 4.4848 | 0.9394 | 1.80E-06 | 0.977 | 1 | 0.911 | rs9493193 |
| 5 | 50062611 | T | C | 0.21 | -1.0011 | 0.2097 | 1.80E-06 | 0.999 | 1 | 0.994 | rs10512906 |
| 22 | 24910182 | T | C | 0.03 | 2.8452 | 0.5961 | 1.81E-06 | 0.998 | 1 | 0.939 | rs116360446 |
| 6 | 132469601 | G | A | 0.26 | 4.4842 | 0.9397 | 1.83E-06 | 0.977 | 1 | 0.911 | rs9493194 |
| 5 | 50065333 | T | C | 0.21 | -1.0007 | 0.2097 | 1.83E-06 | 0.999 | 1 | 0.993 | rs34428476 |
| 5 | 50066049 | C | G | 0.21 | -0.9998 | 0.2097 | 1.87E-06 | 0.999 | 1 | 0.993 | rs1993955 |
| 7 | 12192936 | G | A | 0.02 | 8.3080 | 1.7427 | 1.87E-06 | 0.999 | 1 | 0.957 | rs74893375 |
| 22 | 24894242 | G | A | 0.03 | 2.8411 | 0.5960 | 1.87E-06 | 0.999 | 1 | 0.964 | rs76833164 |
| 6 | 132470166 | C | T | 0.26 | 4.4833 | 0.9409 | 1.89E-06 | 0.976 | 1 | 0.909 | rs9483391 |
| 5 | 50051320 | A | G | 0.21 | -1.0011 | 0.2104 | 1.94E-06 | 0.999 | 1 | 0.994 | rs113240270 |
| 22 | 24889282 | A | G | 0.03 | 2.8373 | 0.5962 | 1.94E-06 | 0.998 | 1 | 0.932 | rs141235460 |
| 13 | 38205450 | G | C | 0.51 | 0.7825 | 0.1644 | 1.94E-06 | 0.983 | 1 | 0.950 | rs9566243 |
| 5 | 50048822 | A | C | 0.21 | -1.0013 | 0.2104 | 1.95E-06 | 0.999 | 1 | 0.993 | rs35328303 |
| 22 | 24830400 | A | C | 0.03 | 2.8364 | 0.5961 | 1.96E-06 | 0.997 | 1 | 0.935 | rs75727533 |
| 22 | 24830401 | A | C | 0.03 | 2.8364 | 0.5961 | 1.96E-06 | 0.997 | 1 | 0.935 | rs80355897 |
| 22 | 24828853 | T | C | 0.06 | 2.8362 | 0.5961 | 1.96E-06 | 1.000 | 1 | 1.000 | rs71651683 |
| 22 | 24827571 | A | G | 0.03 | 2.8360 | 0.5962 | 1.96E-06 | 0.997 | 1 | 0.935 | rs150722843 |
| 22 | 24848603 | C | T | 0.06 | 2.8342 | 0.5960 | 1.98E-06 | 0.999 | 1 | 0.979 | rs4257477 |
| 22 | 24877307 | A | G | 0.06 | 2.8341 | 0.5959 | 1.98E-06 | 0.997 | 1 | 0.961 | rs78088707 |
| 22 | 24844948 | A | G | 0.06 | 2.8342 | 0.5960 | 1.98E-06 | 0.999 | 1 | 0.986 | rs17004922 |
| 22 | 24859898 | T | C | 0.06 | 2.8342 | 0.5960 | 1.98E-06 | 0.997 | 1 | 0.964 | rs113312602 |
| 22 | 24842031 | T | C | 0.06 | 2.8341 | 0.5960 | 1.98E-06 | 0.999 | 1 | 0.990 | rs74813735 |
| 22 | 24842494 | G | A | 0.03 | 2.8341 | 0.5960 | 1.98E-06 | 0.997 | 1 | 0.929 | rs79760227 |
| 22 | 24842684 | T | C | 0.06 | 2.8341 | 0.5960 | 1.98E-06 | 0.999 | 1 | 0.989 | rs78625928 |
| 22 | 24843219 | C | A | 0.03 | 2.8341 | 0.5960 | 1.98E-06 | 0.996 | 1 | 0.911 | rs78663037 |
| 22 | 24854950 | T | C | 0.06 | 2.8338 | 0.5960 | 1.98E-06 | 0.998 | 1 | 0.973 | rs145442727 |
| 5 | 50071254 | T | C | 0.21 | -0.9969 | 0.2097 | 2.01E-06 | 0.998 | 1 | 0.991 | rs62365481 |
| 5 | 50045967 | T | G | 0.63 | 0.8452 | 0.1779 | 2.01E-06 | 0.990 | 1 | 0.968 | rs2442107 |
| 5 | 50072860 | C | T | 0.21 | -0.9959 | 0.2097 | 2.05E-06 | 0.999 | 1 | 0.993 | rs67151831 |
| 6 | 132471611 | A | G | 0.26 | 4.4762 | 0.9429 | 2.06E-06 | 0.976 | 1 | 0.908 | rs1321271 |
| 5 | 50043652 | A | G | 0.21 | -0.9999 | 0.2107 | 2.09E-06 | 0.999 | 1 | 0.993 | rs62368285 |
| 6 | 132471728 | T | G | 0.26 | 4.4740 | 0.9430 | 2.09E-06 | 0.976 | 1 | 0.908 | rs1321273 |
| 9 | 36927319 | T | C | 0.52 | -0.7616 | 0.1609 | 2.21E-06 | 0.975 | 1 | 0.925 | rs7032726 |
| 13 | 38204301 | C | T | 0.38 | -0.7793 | 0.1647 | 2.22E-06 | 0.986 | 1 | 0.958 | rs1924292 |
| 13 | 38204952 | C | G | 0.38 | -0.7793 | 0.1647 | 2.23E-06 | 0.986 | 1 | 0.958 | rs9547987 |
| 5 | 50035910 | A | C | 0.21 | -0.9985 | 0.2111 | 2.25E-06 | 0.999 | 1 | 0.993 | rs34241669 |
| 6 | 132473180 | C | T | 0.25 | 4.4686 | 0.9453 | 2.27E-06 | 0.975 | 1 | 0.905 | rs6937406 |
| 6 | 132473641 | A | C | 0.25 | 4.4709 | 0.9459 | 2.28E-06 | 0.975 | 1 | 0.904 | rs7756259 |
| 6 | 132473821 | A | C | 0.25 | 4.4712 | 0.9460 | 2.29E-06 | 0.975 | 1 | 0.904 | rs7756584 |
| 9 | 36927558 | T | C | 0.52 | -0.7602 | 0.1609 | 2.30E-06 | 0.975 | 1 | 0.925 | rs10758416 |
| 6 | 132473980 | G | A | 0.25 | 4.4707 | 0.9463 | 2.31E-06 | 0.975 | 1 | 0.904 | rs7756735 |
| 5 | 49967825 | A | G | 0.22 | -1.0027 | 0.2124 | 2.34E-06 | 0.998 | 1 | 0.989 | rs13166556 |
| 5 | 49970496 | G | T | 0.21 | -1.0070 | 0.2133 | 2.35E-06 | 0.998 | 1 | 0.990 | rs12653772 |
| 7 | 12190952 | C | T | 0.02 | 8.2094 | 1.7390 | 2.35E-06 | 0.999 | 1 | 0.951 | rs77668661 |
| 6 | 132474150 | T | G | 0.25 | 4.4691 | 0.9469 | 2.36E-06 | 0.975 | 1 | 0.903 | rs7757338 |
| 12 | 76420006 | T | C | 0.12 | -1.1115 | 0.2356 | 2.38E-06 | 0.971 | 1 | 0.791 | rs73133134 |
| 13 | 38197604 | A | C | 0.46 | -0.7748 | 0.1642 | 2.39E-06 | 0.987 | 1 | 0.964 | rs1924307 |
| 10 | 62523410 | G | A | 0.29 | 0.8000 | 0.1699 | 2.49E-06 | 0.967 | 1 | 0.882 | rs10761557 |
| 5 | 50024757 | C | A | 0.21 | -0.9952 | 0.2117 | 2.58E-06 | 0.998 | 1 | 0.992 | rs13162945 |
| 6 | 132475579 | A | T | 0.25 | 4.4624 | 0.9494 | 2.60E-06 | 0.975 | 1 | 0.902 | rs9493197 |
| 6 | 132475689 | C | A | 0.25 | 4.4615 | 0.9498 | 2.64E-06 | 0.975 | 1 | 0.901 | rs9493200 |
| 5 | 49985525 | T | C | 0.21 | -1.0001 | 0.2129 | 2.65E-06 | 0.997 | 1 | 0.987 | rs62366945 |
| 5 | 50087466 | T | G | 0.21 | -0.9887 | 0.2105 | 2.65E-06 | 0.999 | 1 | 0.994 | rs12654399 |
| 5 | 50021795 | A | G | 0.21 | -0.9944 | 0.2118 | 2.67E-06 | 0.998 | 1 | 0.991 | rs35571312 |
| 5 | 50088850 | A | G | 0.21 | -0.9881 | 0.2105 | 2.68E-06 | 0.999 | 1 | 0.995 | rs3806887 |
| 9 | 36928288 | C | T | 0.52 | -0.7540 | 0.1606 | 2.68E-06 | 0.975 | 1 | 0.925 | rs4880038 |
| 5 | 49988990 | A | T | 0.21 | -0.9989 | 0.2128 | 2.69E-06 | 0.997 | 1 | 0.987 | rs61479216 |
| 13 | 38203427 | G | A | 0.38 | -0.7716 | 0.1645 | 2.73E-06 | 0.987 | 1 | 0.959 | rs1924288 |
| 9 | 36928389 | A | C | 0.52 | -0.7530 | 0.1606 | 2.76E-06 | 0.975 | 1 | 0.925 | rs4880039 |
| 13 | 38196693 | T | C | 0.38 | -0.7706 | 0.1645 | 2.81E-06 | 0.986 | 1 | 0.958 | rs4943524 |
| 1 | 207980901 | A | G | 0.12 | 0.9158 | 0.1957 | 2.86E-06 | 0.995 | 1 | 0.966 | rs4844620 |
| 6 | 132476624 | C | T | 0.25 | 4.4519 | 0.9519 | 2.91E-06 | 0.974 | 1 | 0.900 | rs9493201 |
| 19 | 55207385 | A | G | 0.03 | 6.2117 | 1.3283 | 2.92E-06 | 0.990 | 1 | 0.721 | rs115110234 |
| 19 | 55208317 | G | T | 0.03 | 6.2206 | 1.3304 | 2.93E-06 | 0.991 | 1 | 0.728 | rs59717702 |
| 6 | 132476741 | G | A | 0.25 | 4.4522 | 0.9522 | 2.93E-06 | 0.974 | 1 | 0.898 | rs9493202 |
| 19 | 55208035 | A | G | 0.03 | 6.2157 | 1.3297 | 2.95E-06 | 0.991 | 1 | 0.728 | rs60701393 |
| 13 | 87264407 | G | C | 0.88 | -0.9247 | 0.1979 | 2.96E-06 | 0.997 | 1 | 0.974 | rs1343559 |
| 5 | 50007911 | C | A | 0.21 | -0.9918 | 0.2123 | 2.97E-06 | 0.998 | 1 | 0.988 | rs34389644 |
| 8 | 18001540 | G | A | 0.35 | -1.1282 | 0.2415 | 2.98E-06 | 0.989 | 1 | 0.964 | rs6586700 |
| 19 | 55203787 | C | T | 0.03 | 6.1882 | 1.3249 | 3.00E-06 | 0.990 | 1 | 0.713 | rs59013252 |
| 6 | 132477310 | A | G | 0.27 | 4.4549 | 0.9544 | 3.04E-06 | 0.972 | 1 | 0.896 | rs7745229 |
| 6 | 132477049 | G | A | 0.25 | 4.4474 | 0.9530 | 3.06E-06 | 0.974 | 1 | 0.900 | rs9483392 |
| 7 | 12188928 | C | T | 0.02 | 8.0825 | 1.7327 | 3.09E-06 | 0.998 | 1 | 0.932 | rs79233778 |
| 7 | 152650529 | G | A | 0.06 | 1.5078 | 0.3232 | 3.09E-06 | 0.982 | 1 | 0.756 | rs77221864 |
| 5 | 49985652 | G | A | 0.42 | 0.7847 | 0.1682 | 3.10E-06 | 0.997 | 1 | 0.989 | rs11954386 |
| 8 | 18029698 | A | G | 0.15 | -0.9548 | 0.2048 | 3.13E-06 | 0.996 | 1 | 0.975 | rs34403329 |
| 19 | 55202187 | G | A | 0.03 | 6.1762 | 1.3249 | 3.14E-06 | 0.991 | 1 | 0.721 | rs60047340 |
| 13 | 43778315 | A | G | 0.02 | 1.8182 | 0.3901 | 3.15E-06 | 0.995 | 1 | 0.806 | rs138959261 |
| 6 | 132477471 | A | G | 0.27 | 4.4456 | 0.9539 | 3.16E-06 | 0.975 | 1 | 0.907 | rs7745523 |
| 9 | 36926028 | T | C | 0.52 | -0.7500 | 0.1610 | 3.17E-06 | 0.976 | 1 | 0.927 | rs3780151 |
| 9 | 36928662 | G | T | 0.52 | -0.7491 | 0.1608 | 3.18E-06 | 0.975 | 1 | 0.925 | rs10758417 |
| 5 | 49952219 | C | G | 0.22 | -0.9892 | 0.2124 | 3.21E-06 | 0.997 | 1 | 0.985 | rs12654329 |
| 22 | 24821764 | A | G | 0.04 | 2.7733 | 0.5956 | 3.23E-06 | 0.998 | 1 | 0.959 | rs116704292 |
| 1 | 207983745 | C | A | 0.18 | 0.8982 | 0.1930 | 3.27E-06 | 0.998 | 1 | 0.990 | rs66532523 |
| 1 | 207979577 | A | T | 0.18 | 0.8981 | 0.1931 | 3.30E-06 | 0.999 | 1 | 0.993 | rs11118612 |
| 5 | 50144735 | A | C | 0.22 | -0.9676 | 0.2084 | 3.42E-06 | 0.983 | 1 | 0.925 | rs1862547 |
| 22 | 24821154 | T | G | 0.06 | 2.7644 | 0.5954 | 3.44E-06 | 0.999 | 1 | 0.985 | rs138019862 |
| 9 | 36928909 | T | G | 0.52 | -0.7465 | 0.1608 | 3.46E-06 | 0.975 | 1 | 0.925 | rs4878668 |
| 1 | 208028029 | C | T | 0.21 | 0.8902 | 0.1918 | 3.46E-06 | 0.997 | 1 | 0.988 | rs11586197 |
| 5 | 154005654 | G | T | 0.57 | -0.8845 | 0.1907 | 3.50E-06 | 0.971 | 1 | 0.914 | rs7731137 |
| 6 | 132479414 | T | A | 0.27 | 4.4350 | 0.9561 | 3.51E-06 | 0.975 | 1 | 0.906 | rs9493203 |
| 6 | 132479518 | G | A | 0.27 | 4.4316 | 0.9564 | 3.59E-06 | 0.975 | 1 | 0.907 | rs9493204 |
| 10 | 1504987 | G | C | 0.21 | -0.8577 | 0.1851 | 3.61E-06 | 0.984 | 1 | 0.930 | rs11250535 |
| 1 | 207991209 | G | C | 0.18 | 0.8946 | 0.1933 | 3.67E-06 | 0.998 | 1 | 0.988 | rs4844392 |
| 6 | 132480309 | C | T | 0.27 | 4.4324 | 0.9576 | 3.68E-06 | 0.975 | 1 | 0.906 | rs2876171 |
| 6 | 132480318 | G | A | 0.27 | 4.4333 | 0.9579 | 3.69E-06 | 0.975 | 1 | 0.907 | rs2876172 |
| 9 | 36936554 | A | C | 0.53 | -0.7423 | 0.1605 | 3.75E-06 | 0.973 | 1 | 0.924 | rs3780152 |
| 6 | 132480449 | T | A | 0.27 | 4.4277 | 0.9582 | 3.82E-06 | 0.975 | 1 | 0.906 | rs9483393 |
| 5 | 50149333 | A | G | 0.21 | -0.9638 | 0.2086 | 3.83E-06 | 0.984 | 1 | 0.929 | rs62365519 |
| 9 | 36925197 | T | C | 0.51 | -0.7642 | 0.1654 | 3.84E-06 | 0.972 | 1 | 0.918 | rs7851671 |
| 6 | 132481306 | A | T | 0.27 | 4.4234 | 0.9593 | 4.00E-06 | 0.975 | 1 | 0.906 | rs9483394 |
| 6 | 132480985 | C | T | 0.27 | 4.4205 | 0.9587 | 4.01E-06 | 0.975 | 1 | 0.907 | rs9493207 |
| 5 | 49931074 | T | G | 0.22 | -0.9857 | 0.2139 | 4.05E-06 | 0.995 | 1 | 0.975 | rs35540479 |
| 5 | 50152455 | A | G | 0.22 | -0.9605 | 0.2084 | 4.06E-06 | 0.982 | 1 | 0.921 | rs16886818 |
| 8 | 18036227 | C | G | 0.07 | -1.0740 | 0.2331 | 4.08E-06 | 0.997 | 1 | 0.961 | rs17594166 |
| 13 | 38194341 | G | C | 0.51 | -0.7596 | 0.1649 | 4.10E-06 | 0.985 | 1 | 0.956 | rs4941875 |
| 5 | 49927816 | G | A | 0.22 | -0.9855 | 0.2141 | 4.16E-06 | 0.995 | 1 | 0.974 | rs13190380 |
| 22 | 24870527 | G | A | 0.11 | 2.6972 | 0.5860 | 4.17E-06 | 0.996 | 1 | 0.972 | rs17842490 |
| 22 | 24878741 | T | C | 0.11 | 2.6972 | 0.5860 | 4.17E-06 | 0.996 | 1 | 0.972 | rs1547359 |
| 22 | 24878110 | C | T | 0.11 | 2.6970 | 0.5860 | 4.18E-06 | 0.996 | 1 | 0.972 | rs57452279 |
| 13 | 38099921 | A | C | 0.39 | -0.7355 | 0.1600 | 4.27E-06 | 0.977 | 1 | 0.925 | rs9315498 |
| 5 | 49920921 | T | C | 0.21 | -0.9833 | 0.2140 | 4.32E-06 | 0.994 | 1 | 0.970 | rs35749852 |
| 1 | 208035434 | A | T | 0.20 | 0.8844 | 0.1925 | 4.33E-06 | 0.992 | 1 | 0.966 | rs4844623 |
| 6 | 132478286 | T | C | 0.27 | 4.3750 | 0.9522 | 4.33E-06 | 0.974 | 1 | 0.905 | rs7356898 |
| 1 | 208037821 | T | C | 0.21 | 0.8844 | 0.1925 | 4.34E-06 | 0.995 | 1 | 0.977 | rs12145290 |
| 1 | 208035446 | G | A | 0.20 | 0.8843 | 0.1925 | 4.34E-06 | 0.992 | 1 | 0.966 | rs4844624 |
| 5 | 49915907 | A | T | 0.28 | -0.9815 | 0.2139 | 4.46E-06 | 0.994 | 1 | 0.976 | rs34426073 |
| 12 | 50159398 | A | G | 0.07 | 7.0979 | 1.5475 | 4.51E-06 | 0.988 | 1 | 0.870 | rs114109920 |
| 5 | 49993482 | C | T | 0.42 | 0.7673 | 0.1675 | 4.60E-06 | 0.997 | 1 | 0.991 | rs12186752 |
| 1 | 207975949 | C | T | 0.18 | 0.8856 | 0.1933 | 4.61E-06 | 0.999 | 1 | 0.992 | rs56075814 |
| 5 | 50114421 | T | C | 0.21 | -0.9566 | 0.2090 | 4.72E-06 | 0.992 | 1 | 0.961 | rs1125916 |
| 5 | 50127289 | T | A | 0.21 | -0.9563 | 0.2090 | 4.74E-06 | 0.989 | 1 | 0.945 | rs13186089 |
| 5 | 49962166 | A | T | 0.42 | 0.7684 | 0.1680 | 4.80E-06 | 0.999 | 1 | 0.997 | rs66912405 |
| 5 | 49989701 | G | A | 0.42 | 0.7662 | 0.1676 | 4.82E-06 | 0.997 | 1 | 0.991 | rs12055210 |
| 5 | 50024668 | G | A | 0.38 | 0.7623 | 0.1668 | 4.84E-06 | 0.998 | 1 | 0.993 | rs2594708 |
| 5 | 50023374 | A | C | 0.38 | 0.7620 | 0.1668 | 4.88E-06 | 0.998 | 1 | 0.993 | rs10737958 |
| 6 | 132484105 | C | A | 0.27 | 4.3991 | 0.9628 | 4.90E-06 | 0.974 | 1 | 0.905 | rs9321326 |
| 6 | 132484124 | C | T | 0.27 | 4.3990 | 0.9630 | 4.92E-06 | 0.974 | 1 | 0.905 | rs10214443 |
| 5 | 50026928 | T | A | 0.42 | 0.7613 | 0.1667 | 4.95E-06 | 0.997 | 1 | 0.990 | rs204915 |
| 5 | 50025690 | C | G | 0.38 | 0.7611 | 0.1668 | 5.01E-06 | 0.998 | 1 | 0.993 | rs2463796 |
| 9 | 36928576 | C | T | 0.52 | -0.7331 | 0.1606 | 5.02E-06 | 0.976 | 1 | 0.927 | rs4878667 |
| 5 | 50026465 | T | C | 0.42 | 0.7607 | 0.1667 | 5.06E-06 | 0.997 | 1 | 0.990 | rs282544 |
| 10 | 62524139 | C | T | 0.27 | 0.7777 | 0.1706 | 5.12E-06 | 0.968 | 1 | 0.883 | rs10761559 |
| 3 | 133816175 | A | G | 0.01 | 5.8536 | 1.2838 | 5.13E-06 | 0.997 | 1 | 0.830 | rs144714360 |
| 10 | 62544179 | T | C | 0.28 | 0.7868 | 0.1726 | 5.13E-06 | 0.974 | 1 | 0.910 | rs3213045 |
| 6 | 132484635 | G | A | 0.27 | 4.3946 | 0.9640 | 5.14E-06 | 0.975 | 1 | 0.906 | rs9493210 |
| 5 | 50032285 | A | G | 0.43 | 0.7593 | 0.1666 | 5.16E-06 | 0.995 | 1 | 0.985 | rs282556 |
| 5 | 49978833 | A | G | 0.42 | 0.7650 | 0.1679 | 5.20E-06 | 0.998 | 1 | 0.994 | rs36077571 |
| 5 | 50037886 | T | C | 0.43 | 0.7580 | 0.1665 | 5.29E-06 | 0.995 | 1 | 0.985 | rs282560 |
| 5 | 49983137 | C | G | 0.42 | 0.7637 | 0.1678 | 5.30E-06 | 0.998 | 1 | 0.993 | rs55932149 |
| 5 | 50037451 | G | A | 0.43 | 0.7578 | 0.1665 | 5.33E-06 | 0.995 | 1 | 0.985 | rs166863 |
| 22 | 24784051 | T | A | 0.15 | 2.5189 | 0.5535 | 5.34E-06 | 0.997 | 1 | 0.979 | rs5760389 |
| 5 | 50014674 | G | A | 0.38 | 0.7603 | 0.1671 | 5.37E-06 | 0.998 | 1 | 0.992 | rs1375128 |
| 5 | 49971937 | A | T | 0.42 | 0.7644 | 0.1680 | 5.39E-06 | 0.999 | 1 | 0.995 | rs2354533 |
| 19 | 56229834 | C | T | 0.07 | -1.2962 | 0.2851 | 5.46E-06 | 0.978 | 1 | 0.736 | rs77378270 |
| 2 | 158855781 | T | C | 0.08 | 1.8813 | 0.4138 | 5.47E-06 | 0.994 | 1 | 0.934 | rs116430246 |
| 10 | 62546089 | T | G | 0.28 | 0.7836 | 0.1725 | 5.54E-06 | 0.974 | 1 | 0.913 | rs12220787 |
| 1 | 208030303 | G | A | 0.17 | 0.8709 | 0.1918 | 5.57E-06 | 0.999 | 1 | 0.993 | rs4844621 |
| 1 | 208029947 | T | C | 0.16 | 0.8709 | 0.1918 | 5.61E-06 | 1.000 | 1 | 1.000 | rs7550821 |
| 1 | 208037967 | A | G | 0.10 | 0.9012 | 0.1985 | 5.65E-06 | 0.997 | 1 | 0.975 | rs4844395 |
| 5 | 50139358 | T | A | 0.42 | 0.7530 | 0.1660 | 5.70E-06 | 0.981 | 1 | 0.941 | rs27243 |
| 1 | 207959070 | C | T | 0.11 | 0.8758 | 0.1931 | 5.73E-06 | 0.996 | 1 | 0.972 | rs11118580 |
| 12 | 49994658 | G | C | 0.07 | 6.9360 | 1.5295 | 5.76E-06 | 0.993 | 1 | 0.923 | rs74086906 |
| 5 | 50056435 | G | A | 0.42 | 0.7530 | 0.1661 | 5.77E-06 | 0.997 | 1 | 0.991 | rs282548 |
| 19 | 56229833 | C | T | 0.07 | -1.2934 | 0.2854 | 5.85E-06 | 0.978 | 1 | 0.734 | rs74421704 |
| 1 | 208027696 | T | C | 0.17 | 0.8699 | 0.1920 | 5.89E-06 | 0.998 | 1 | 0.988 | rs61821315 |
| 6 | 92915192 | T | G | 0.16 | -0.9506 | 0.2099 | 5.95E-06 | 0.996 | 1 | 0.975 | rs1596489 |
| 8 | 18000802 | A | G | 0.08 | -1.1295 | 0.2495 | 5.97E-06 | 0.992 | 1 | 0.919 | rs113619473 |
| 5 | 49964905 | C | T | 0.42 | 0.7622 | 0.1684 | 6.04E-06 | 0.999 | 1 | 0.995 | rs61401688 |
| 5 | 49950279 | T | C | 0.42 | 0.7598 | 0.1680 | 6.08E-06 | 0.998 | 1 | 0.992 | rs2883460 |
| 5 | 49960673 | C | A | 0.43 | 0.7597 | 0.1680 | 6.10E-06 | 1.000 | 1 | 0.999 | rs10471414 |
| 5 | 50063035 | T | G | 0.58 | -0.7502 | 0.1659 | 6.12E-06 | 0.996 | 1 | 0.988 | rs2404958 |
| 6 | 92917737 | A | G | 0.21 | -0.9476 | 0.2097 | 6.19E-06 | 0.999 | 1 | 0.996 | rs10944572 |
| 6 | 92917834 | A | T | 0.21 | -0.9475 | 0.2097 | 6.20E-06 | 0.999 | 1 | 0.996 | rs12212351 |
| 5 | 50064387 | C | T | 0.58 | -0.7497 | 0.1659 | 6.20E-06 | 0.996 | 1 | 0.988 | rs6450391 |
| 5 | 49982726 | A | T | 0.38 | 0.7584 | 0.1678 | 6.20E-06 | 0.998 | 1 | 0.992 | rs7717033 |
| 6 | 92917957 | A | C | 0.21 | -0.9475 | 0.2097 | 6.20E-06 | 0.999 | 1 | 0.996 | rs7744502 |
| 6 | 92917417 | A | G | 0.21 | -0.9476 | 0.2097 | 6.20E-06 | 0.999 | 1 | 0.994 | rs7740007 |
| 6 | 92918248 | C | T | 0.21 | -0.9474 | 0.2097 | 6.22E-06 | 0.999 | 1 | 0.997 | rs1444134 |
| 6 | 92918288 | A | G | 0.21 | -0.9474 | 0.2096 | 6.22E-06 | 1.000 | 1 | 0.997 | rs1444133 |
| 6 | 92919470 | G | A | 0.16 | -0.9483 | 0.2099 | 6.22E-06 | 0.997 | 1 | 0.980 | rs12193981 |
| 2 | 169386399 | T | C | 0.01 | 6.6620 | 1.4744 | 6.23E-06 | 0.999 | 1 | 0.959 | rs78436266 |
| 6 | 92918457 | C | T | 0.21 | -0.9472 | 0.2096 | 6.24E-06 | 0.999 | 1 | 0.996 | rs1444132 |
| 2 | 158864060 | G | A | 0.08 | 1.8850 | 0.4172 | 6.24E-06 | 0.992 | 1 | 0.917 | rs34617636 |
| 6 | 92922779 | C | T | 0.16 | -0.9492 | 0.2101 | 6.26E-06 | 0.995 | 1 | 0.971 | rs12210356 |
| 6 | 92918990 | T | C | 0.21 | -0.9468 | 0.2096 | 6.29E-06 | 0.999 | 1 | 0.994 | rs996731 |
| 6 | 92919082 | G | A | 0.21 | -0.9468 | 0.2096 | 6.29E-06 | 0.999 | 1 | 0.993 | rs996730 |
| 6 | 92919277 | T | A | 0.21 | -0.9467 | 0.2096 | 6.30E-06 | 0.999 | 1 | 0.993 | rs12193871 |
| 6 | 92919666 | T | G | 0.21 | -0.9463 | 0.2096 | 6.35E-06 | 0.998 | 1 | 0.990 | rs12207640 |
| 6 | 92919804 | T | C | 0.21 | -0.9463 | 0.2096 | 6.36E-06 | 0.998 | 1 | 0.990 | rs12207608 |
| 6 | 92919958 | T | C | 0.21 | -0.9461 | 0.2096 | 6.38E-06 | 0.998 | 1 | 0.989 | rs12198969 |
| 6 | 92920109 | T | C | 0.21 | -0.9459 | 0.2096 | 6.40E-06 | 0.998 | 1 | 0.988 | rs10944574 |
| 5 | 50058684 | A | C | 0.58 | -0.7486 | 0.1659 | 6.43E-06 | 0.997 | 1 | 0.990 | rs6450384 |
| 22 | 24902412 | G | A | 0.05 | 2.6480 | 0.5869 | 6.44E-06 | 0.996 | 1 | 0.933 | rs61228157 |
| 8 | 18027610 | C | G | 0.07 | -1.1221 | 0.2487 | 6.45E-06 | 0.997 | 1 | 0.967 | rs73587955 |
| 1 | 207934849 | G | A | 0.12 | 0.8694 | 0.1927 | 6.46E-06 | 0.997 | 1 | 0.976 | rs4844390 |
| 1 | 207960708 | T | C | 0.19 | 0.8694 | 0.1927 | 6.46E-06 | 0.998 | 1 | 0.991 | rs4844619 |
| 10 | 62533306 | T | C | 0.24 | 0.7819 | 0.1734 | 6.48E-06 | 0.972 | 1 | 0.894 | rs1904412 |
| 3 | 133943346 | A | G | 0.01 | 5.8848 | 1.3053 | 6.53E-06 | 0.998 | 1 | 0.848 | rs116931000 |
| 22 | 24899350 | G | T | 0.05 | 2.6462 | 0.5870 | 6.54E-06 | 0.996 | 1 | 0.936 | rs114445986 |
| 22 | 24900359 | G | A | 0.05 | 2.6456 | 0.5870 | 6.56E-06 | 0.996 | 1 | 0.935 | rs116812922 |
| 22 | 24907698 | G | C | 0.05 | 2.6454 | 0.5869 | 6.57E-06 | 0.996 | 1 | 0.933 | rs145610981 |
| 22 | 24910706 | G | A | 0.05 | 2.6443 | 0.5869 | 6.62E-06 | 0.996 | 1 | 0.932 | rs16978612 |
| 5 | 49899097 | T | C | 0.28 | -0.9699 | 0.2153 | 6.62E-06 | 0.992 | 1 | 0.968 | rs6897477 |
| 6 | 92923980 | T | G | 0.16 | -0.9449 | 0.2098 | 6.65E-06 | 0.995 | 1 | 0.970 | rs1154604 |
| 5 | 49926200 | A | G | 0.38 | 0.7610 | 0.1689 | 6.66E-06 | 0.994 | 1 | 0.980 | rs6864477 |
| 2 | 169379980 | A | G | 0.01 | 6.6447 | 1.4752 | 6.66E-06 | 0.999 | 1 | 0.955 | rs76173963 |
| 5 | 49944102 | A | G | 0.42 | 0.7581 | 0.1683 | 6.69E-06 | 0.997 | 1 | 0.988 | rs62366910 |
| 5 | 50103818 | A | G | 0.21 | -0.9420 | 0.2092 | 6.72E-06 | 0.995 | 1 | 0.975 | rs16877029 |
| 5 | 50160386 | A | C | 0.41 | 0.7424 | 0.1649 | 6.73E-06 | 0.974 | 1 | 0.918 | rs152001 |
| 1 | 207960104 | T | G | 0.19 | 0.8673 | 0.1927 | 6.81E-06 | 0.999 | 1 | 0.992 | rs6657476 |
| 5 | 49924084 | C | G | 0.42 | 0.7598 | 0.1689 | 6.85E-06 | 0.994 | 1 | 0.981 | rs55849844 |
| 3 | 133871822 | G | A | 0.01 | 5.7064 | 1.2688 | 6.88E-06 | 1.000 | 1 | 0.994 | rs114315063 |
| 5 | 49940710 | G | A | 0.42 | 0.7578 | 0.1685 | 6.88E-06 | 0.996 | 1 | 0.986 | rs72753742 |
| 5 | 49943977 | T | C | 0.42 | 0.7574 | 0.1684 | 6.88E-06 | 0.997 | 1 | 0.987 | rs62366909 |
| 5 | 49943923 | T | A | 0.42 | 0.7574 | 0.1684 | 6.88E-06 | 0.997 | 1 | 0.987 | rs62366908 |
| 1 | 207917499 | G | A | 0.81 | -0.8618 | 0.1917 | 6.92E-06 | 0.997 | 1 | 0.983 | rs2761434 |
| 5 | 49945474 | T | C | 0.38 | 0.7570 | 0.1685 | 7.00E-06 | 0.996 | 1 | 0.986 | rs3864235 |
| 5 | 49931489 | A | G | 0.42 | 0.7584 | 0.1688 | 7.01E-06 | 0.995 | 1 | 0.984 | rs1968638 |
| 3 | 133874216 | C | A | 0.01 | 5.7023 | 1.2694 | 7.06E-06 | 0.999 | 1 | 0.947 | rs74474984 |
| 8 | 16545754 | T | C | 0.13 | 9.6574 | 2.1509 | 7.12E-06 | 0.989 | 1 | 0.927 | rs113119941 |
| 8 | 16545780 | T | C | 0.13 | 9.6574 | 2.1509 | 7.12E-06 | 0.989 | 1 | 0.928 | rs17123471 |
| 6 | 132487698 | T | A | 0.27 | 4.3514 | 0.9691 | 7.12E-06 | 0.974 | 1 | 0.903 | rs6940428 |
| 10 | 62548718 | A | G | 0.26 | 0.7734 | 0.1723 | 7.13E-06 | 0.973 | 1 | 0.904 | rs3213058 |
| 5 | 50071565 | G | A | 0.57 | -0.7448 | 0.1659 | 7.14E-06 | 0.994 | 1 | 0.980 | rs154136 |
| 6 | 132487723 | C | T | 0.27 | 4.3507 | 0.9692 | 7.15E-06 | 0.974 | 1 | 0.903 | rs6920169 |
| 5 | 49929456 | A | G | 0.42 | 0.7579 | 0.1689 | 7.17E-06 | 0.995 | 1 | 0.983 | rs28690842 |
| 1 | 208030856 | T | C | 0.16 | 0.8644 | 0.1927 | 7.25E-06 | 0.999 | 1 | 0.994 | rs7551724 |
| 1 | 208031234 | A | G | 0.16 | 0.8644 | 0.1927 | 7.25E-06 | 0.998 | 1 | 0.990 | rs12141901 |
| 1 | 208033706 | T | C | 0.16 | 0.8643 | 0.1927 | 7.26E-06 | 0.997 | 1 | 0.985 | rs61821318 |
| 2 | 169399760 | A | G | 0.01 | 6.6379 | 1.4799 | 7.28E-06 | 1.000 | 1 | 0.977 | rs77248493 |
| 1 | 208036509 | T | C | 0.16 | 0.8643 | 0.1927 | 7.28E-06 | 0.996 | 1 | 0.979 | rs882198 |
| 1 | 207920788 | T | C | 0.81 | -0.8589 | 0.1915 | 7.32E-06 | 0.998 | 1 | 0.991 | rs2796265 |
| 1 | 207923081 | G | A | 0.81 | -0.8592 | 0.1916 | 7.32E-06 | 0.997 | 1 | 0.985 | rs2761437 |
| 5 | 50152467 | C | T | 0.38 | 0.7433 | 0.1658 | 7.32E-06 | 0.980 | 1 | 0.935 | rs27964 |
| 5 | 50121623 | T | C | 0.58 | -0.7435 | 0.1658 | 7.32E-06 | 0.984 | 1 | 0.950 | rs27255 |
| 6 | 132487810 | G | C | 0.27 | 4.3460 | 0.9694 | 7.36E-06 | 0.974 | 1 | 0.903 | rs6940746 |
| 6 | 132488116 | A | G | 0.26 | 4.3511 | 0.9706 | 7.37E-06 | 0.971 | 1 | 0.892 | rs6941580 |
| 2 | 175093901 | G | C | 0.15 | 0.8824 | 0.1969 | 7.42E-06 | 0.989 | 1 | 0.937 | rs6752755 |
| 10 | 62550014 | G | A | 0.28 | 0.7728 | 0.1726 | 7.56E-06 | 0.974 | 1 | 0.913 | rs3213067 |
| 1 | 208034329 | T | C | 0.16 | 0.8627 | 0.1928 | 7.63E-06 | 0.997 | 1 | 0.982 | rs4844622 |
| 1 | 72302396 | T | G | 0.02 | -1.8605 | 0.4160 | 7.73E-06 | 0.999 | 1 | 0.942 | rs1426177 |
| 6 | 132489527 | G | A | 0.27 | 4.3362 | 0.9709 | 7.97E-06 | 0.972 | 1 | 0.899 | rs6569775 |
| 2 | 175099237 | A | G | 0.15 | 0.8870 | 0.1987 | 8.02E-06 | 0.990 | 1 | 0.938 | rs56094369 |
| 2 | 175105204 | A | T | 0.23 | 0.8846 | 0.1982 | 8.03E-06 | 0.986 | 1 | 0.940 | rs35735114 |
| 3 | 193593314 | C | T | 0.05 | 1.6079 | 0.3602 | 8.06E-06 | 0.990 | 1 | 0.824 | rs79773931 |
| 1 | 207934487 | C | A | 0.80 | -0.8582 | 0.1923 | 8.09E-06 | 0.999 | 1 | 0.993 | rs2466572 |
| 1 | 207930203 | A | G | 0.82 | -0.8573 | 0.1921 | 8.11E-06 | 1.000 | 1 | 0.999 | rs2724384 |
| 8 | 74628306 | C | T | 0.12 | -0.9193 | 0.2060 | 8.12E-06 | 0.979 | 1 | 0.852 | rs55639005 |
| 2 | 175102804 | A | G | 0.15 | 0.8859 | 0.1985 | 8.13E-06 | 0.990 | 1 | 0.940 | rs12999101 |
| 10 | 62525424 | T | C | 0.24 | 0.7734 | 0.1734 | 8.18E-06 | 0.971 | 1 | 0.887 | rs10994503 |
| 1 | 207941191 | T | G | 0.80 | -0.8583 | 0.1924 | 8.18E-06 | 0.998 | 1 | 0.990 | rs2724374 |
| 1 | 207943158 | T | C | 0.80 | -0.8584 | 0.1925 | 8.22E-06 | 0.998 | 1 | 0.989 | rs2724360 |
| 5 | 49917974 | T | A | 0.28 | -0.9615 | 0.2157 | 8.25E-06 | 0.992 | 1 | 0.969 | rs62366899 |
| 2 | 175106028 | A | C | 0.16 | 0.8848 | 0.1985 | 8.28E-06 | 0.987 | 1 | 0.927 | rs13424932 |
| 5 | 50106439 | A | G | 0.62 | -0.7388 | 0.1659 | 8.42E-06 | 0.991 | 1 | 0.967 | rs32396 |
| 5 | 50044622 | C | G | 0.42 | 0.7438 | 0.1670 | 8.48E-06 | 0.995 | 1 | 0.983 | rs2463797 |
| 10 | 62523470 | A | G | 0.26 | 0.7596 | 0.1706 | 8.49E-06 | 0.969 | 1 | 0.885 | rs10761558 |
| 5 | 49912509 | G | A | 0.42 | 0.7526 | 0.1691 | 8.52E-06 | 0.993 | 1 | 0.978 | rs11746825 |
| 9 | 26190219 | C | T | 0.55 | -0.7368 | 0.1655 | 8.56E-06 | 0.988 | 1 | 0.967 | rs10812360 |
| 13 | 38248655 | G | A | 0.46 | -0.7328 | 0.1647 | 8.68E-06 | 0.982 | 1 | 0.948 | rs1924304 |
| 6 | 132490942 | T | C | 0.27 | 4.3174 | 0.9725 | 9.01E-06 | 0.973 | 1 | 0.899 | rs6916021 |
| 1 | 208039471 | C | G | 0.16 | 0.8572 | 0.1931 | 9.06E-06 | 0.993 | 1 | 0.965 | rs1967689 |
| 5 | 50147620 | A | G | 0.21 | -0.9226 | 0.2080 | 9.21E-06 | 0.985 | 1 | 0.931 | rs12659764 |
| 22 | 24814545 | A | G | 0.06 | 2.6133 | 0.5895 | 9.27E-06 | 0.998 | 1 | 0.974 | rs114269033 |
| 4 | 97762056 | T | C | 0.01 | -3.3036 | 0.7456 | 9.40E-06 | 0.996 | 1 | 0.777 | rs142979224 |
| 5 | 49887903 | C | T | 0.38 | 0.7557 | 0.1706 | 9.41E-06 | 0.991 | 1 | 0.970 | rs10447119 |
| 5 | 49958605 | T | C | 0.41 | 0.7425 | 0.1676 | 9.42E-06 | 0.998 | 1 | 0.992 | rs12153387 |
| 1 | 208007277 | G | T | 0.18 | 0.8608 | 0.1948 | 9.93E-06 | 0.999 | 1 | 0.994 | rs61821293 |
| 22 | 24814232 | G | C | 0.04 | 2.6020 | 0.5889 | 9.94E-06 | 0.997 | 1 | 0.949 | rs114580516 |
| 6 | 92923600 | G | A | 0.30 | -0.9188 | 0.2080 | 9.96E-06 | 0.983 | 1 | 0.942 | rs2218418 |

eTable 3. Leading SNPs from Cortical Clock GWAS in ROSMAP, controlling for neuron proportion

| SNP ID | Chr:pos | Beta Estimate | SE | p-value |
| --- | --- | --- | --- | --- |
| rs4721030 | 7:12137067 | 7.765 | 1.478 | 2.00E-07 |
| rs13180000 | 5:50132506 | -0.992 | 0.200 | 8.50E-07 |
| Rs3788369 | 22:24892973 | 2.686 | 0.592 | 6.73E-06 |
| Rs11998660 | 8:16546686 | 8.667 | 1.750 | 9.19E-07 |
| Rs12386903 | 8:18000966 | -1.255 | 0.242 | 2.69E-07 |
| Rs4943525 | 13:38206239 | 0.794 | 0.163 | 1.33E-06 |
| rs6925799 | 6:132467591 | 4.502 | 0.936 | 1.83E-06 |
| rs7032313 | 9:36927081 | -0.695 | 0.160 | 1.73E-05 |
| rs73133134 | 12:76420006 | -0.951 | 0.213 | 9.79E-06 |
| rs10761557 | 10:62523410 | 0.822 | 0.168 | 1.29E-06 |
| rs4844620 | 1:207980901 | 0.902 | 0.193 | 3.51E-06 |
| rs1343559 | 13:87264407 | -0.888 | 0.195 | 6.53E-06 |
| rs77221864 | 7:152650529 | 0.958 | 0.301 | 0.001525 |
| rs34403329 | 8:18029698 | -1.021 | 0.203 | 6.21E-07 |
| rs7731137 | 5:154005654 | -0.849 | 0.187 | 6.97E-06 |
| rs11250535 | 10:1504987 | -0.639 | 0.169 | 0.000167 |
| rs9315498 | 13:38099921 | -0.683 | 0.159 | 1.97E-05 |
| rs114109920 | 12:50159398 | 6.704 | 1.525 | 1.28E-05 |
| rs77378270 | 19:56229834 | -0.778 | 0.236 | 0.001001 |
| rs116430246 | 2:158855781 | 1.690 | 0.406 | 3.46E-05 |
| rs1596489 | 6:92915192 | -0.966 | 0.208 | 3.98E-06 |
| rs6752755 | 2:175093901 | 0.821 | 0.194 | 2.61E-05 |
| rs55639005 | 8:74628306 | -0.782 | 0.193 | 5.57E-05 |
| rs10812360 | 9:26190219 | -0.720 | 0.162 | 1.06E-05 |

eTable 4. Leading SNPs from Cortical Clock GWAS in ROSMAP, controlling for seven cell type proportions*

| SNP ID | Chr:pos | Beta Estimate | SE | p-value |
| --- | --- | --- | --- | --- |
| rs4721030 | 7:12137067 | 6.731 | 1.391 | 1.65E-06 |
| rs13180000 | 5:50132506 | -0.842 | 0.190 | 1.08E-05 |
| Rs3788369 | 22:24892973 | 2.053 | 0.563 | 2.87E-04 |
| Rs11998660 | 8:16546686 | 8.069 | 1.64 | 1.13E-06 |
| Rs12386903 | 8:18000966 | -0.967 | 0.232 | 3.43E-05 |
| Rs4943525 | 13:38206239 | 0.725 | 0.155 | 3.41E-06 |
| rs6925799 | 6:132467591 | 3.451 | 0.931 | 2.28E-04 |
| rs7032313 | 9:36927081 | -0.640 | 0.154 | 3.49E-05 |
| rs73133134 | 12:76420006 | -0.839 | 0.204 | 4.40E-05 |
| rs10761557 | 10:62523410 | 0.740 | 0.161 | 5.05E-06 |
| rs4844620 | 1:207980901 | 0.754 | 0.184 | 4.59E-05 |
| rs1343559 | 13:87264407 | -0.534 | 0.190 | 5.15E-03 |
| rs77221864 | 7:152650529 | 0.759 | 0.284 | 0.00770 |
| rs34403329 | 8:18029698 | -0.826 | 0.195 | 2.53E-05 |
| rs7731137 | 5:154005654 | -0.700 | 0.179 | 1.05E-04 |
| rs11250535 | 10:1504987 | -0.554 | 0.160 | 0.000585 |
| rs9315498 | 13:38099921 | -0.604 | 0.152 | 8.26E-05 |
| rs114109920 | 12:50159398 | 6.042 | 1.430 | 2.71E-05 |
| rs77378270 | 19:56229834 | -0.566 | 0.225 | 0.0121 |
| rs116430246 | 2:158855781 | 1.239 | 0.392 | 1.65E-03 |
| rs1596489 | 6:92915192 | -0.829 | 0.199 | 3.40E-05 |
| rs6752755 | 2:175093901 | 0.741 | 0.185 | 6.85E-05 |
| rs55639005 | 8:74628306 | -0.613 | 0.185 | 9.83E-04 |
| rs10812360 | 9:26190219 | -0.627 | 0.154 | 5.33E-05 |

*Cell type proportions: inhibitory neurons, excitatory neurons, astrocytes, endothelial cells, oligodendrocytes, oligodendrocyte precursor cells, microglia. Cell type proportions estimated using single cell methylation sequencing as the reference and the Houseman deconvolution algorithm, see ref. 20.

eTable 5. Expression quantitative trait loci from single nucleus RNA-seq in dorsolateral prefrontal cortex (n=424)

| SNP ID | Gene | Beta | FDR p-value | Cell type |
| --- | --- | --- | --- | --- |
| rs11250535 | LARP4B | 0.265 | 0.024 | Endothelial |
| rs34403329 | AC027117.2 | 0.200 | 0.032 | Astrocyte |
| rs4844620 | CD46 | -0.839 | 1.13E-42 | Oligodendrocyte |
| rs4844620 | CD46 | -0.380 | 8.23E-07 | Astrocyte |
| rs4844620 | CD46 | -0.827 | 1.25E-29 | Oligodendrocyte precursor cell |
| rs4844620 | CD46 | -0.561 | 2.52E-17 | Inhibitory neuron |
| rs4844620 | CD46 | -0.579 | 6.71E-26 | Excitatory neuron |
| rs4844620 | CD46 | -0.299 | 0.017 | Microglia |
| rs4844620 | MIR29B2CHG | -0.209 | 0.039 | Microglia |
| rs55639005 | AC027018.1 | -0.204 | 0.000589 | Oligodendrocyte |
| rs55639005 | AC027018.1 | -0.170 | 0.00025 | Astrocyte |
| rs55639005 | AC027018.1 | -0.215 | 0.00285 | Oligodendrocyte precursor cell |
| rs55639005 | AC027018.1 | -0.118 | 0.00428 | Inhibitory neuron |
| rs55639005 | AC027018.1 | -0.144 | 1.58E-06 | Excitatory neuron |
| rs55639005 | STAU2 | -0.118 | 0.00945 | Excitatory neuron |
| rs73133134 | AC078923.1 | -0.582 | 1.63E-12 | Inhibitory neuron |
| rs73133134 | AC078923.1 | -0.674 | 1.03E-21 | Excitatory neuron |
| rs77378270 | EPN1 | 0.338 | 0.0145 | Oligodendrocyte precursor cell |

eTable 6. Enrichment for biologic processes in GO and KEGG

| Term ID | Term name | P-value | intersection |
| --- | --- | --- | --- |
| KEGG:00983 | Drug metabolism - other enzymes | 0.009 | UPP2,UPB1,NAT1 |
| GO:0046135 | pyrimidine nucleoside catabolic process | 0.043 | UPP2,UPB1 |

eTable 7. Relation of Leading SNPs to Aging Phenotypes (for SNPs which were related to an aging phenotype, at nominal significance)

| **Chr:pos** | **beta/OR** | **SE of mean** | **LCI** | **UCI** | **p-value** | **Phenotype** | **rsid** |
| --- | --- | --- | --- | --- | --- | --- | --- |
| 13:100580750 | 0.0408 | 0.0144 | 0.069024 | 0.012576 | 0.0047 | global AD pathology | rs9557340 |
|  | 0.0067 | 0.0027 | 0.011992 | 0.001408 | 0.014 | amyloid deposition |  |
|  | 0.1085 | 0.0353 | 0.177688 | 0.039312 | 0.00217 | tangle density |  |
|  | 1.12 |  | 0.97 | 1.31 | 0.13 | atherosclerosis |  |
|  | 1.06 |  | 0.91 | 1.24 | 0.45 | arteriol |  |
|  | 1.17 |  | 1.03 | 1.32 | 0.0161 | dementia |  |
|  | -0.099 | 0.0403 | -0.02001 | -0.17799 | 0.0138 | baseline global cognition |  |
|  | -0.0101 | 0.0035 | -0.00324 | -0.01696 | 0.0038 | global cognition slope |  |
|  | -0.0047 | 0.009 | 0.01294 | -0.02234 | 0.6038 | baseline motor |  |
|  | 0 | 0.0009 | 0.001764 | -0.00176 | 0.99 | motor slope |  |
|  | -0.0295 | 0.0489 | 0.066344 | -0.12534 | 0.55 | baseline parkinsonism |  |
|  | -0.0072 | 0.0046 | 0.001816 | -0.01622 | 0.1217 | parkinsonism slope |  |
| 1:94646439 | 0.0425 | 0.0296 | 0.100516 | -0.01552 | 0.15 | global AD pathology | rs17111206 |
|  | 0.0066 | 0.0056 | 0.017576 | -0.00438 | 0.23 | amyloid deposition |  |
|  | 0.0582 | 0.0723 | 0.199908 | -0.08351 | 0.42 | tangle density |  |
|  | 0.88 |  | 0.64 | 1.21 | 0.42 | atherosclerosis |  |
|  | 0.69 |  | 0.49 | 0.98 | 0.037 | arteriol |  |
|  | 1.29 |  | 0.99 | 1.66 | 0.0559 | dementia |  |
|  | -0.1428 | 0.0811 | 0.016156 | -0.30176 | 0.0784 | baseline global cognition |  |
|  | -0.007 | 0.0071 | 0.006916 | -0.02092 | 0.33 | global cognition slope |  |
|  | -0.0076 | 0.0179 | 0.027484 | -0.04268 | 0.67 | baseline motor |  |
|  | -0.0002 | 0.0017 | 0.003132 | -0.00353 | 0.9 | motor slope |  |
|  | 0.11 | 0.0988 | 0.303648 | -0.08365 | 0.27 | baseline parkinsonism |  |
|  | 0.0126 | 0.0093 | 0.030828 | -0.00563 | 0.179 | parkinsonism slope |  |
| 17:10080837 | 0.0178 | 0.0188 | 0.054648 | -0.01905 | 0.34 | global AD pathology | rs177443504 |
|  | 0.0019 | 0.0036 | 0.008956 | -0.00516 | 0.59 | amyloid deposition |  |
|  | 0.0799 | 0.0462 | 0.170452 | -0.01065 | 0.084 | tangle density |  |
|  | 1.22 |  | 1.0014 | 1.48 | 0.048 | atherosclerosis |  |
|  | 1.02 |  | 0.84 | 1.25 | 0.83 | arteriol |  |
|  | 1.13 |  | 0.96 | 1.34 | 0.139 | dementia |  |
|  | -0.1523 | 0.0527 | -0.04901 | -0.25559 | 0.0039 | baseline global cognition |  |
|  | -0.0133 | 0.0046 | -0.00428 | -0.02232 | 0.0036 | global cognition slope |  |
|  | 0.0002 | 0.0116 | 0.022936 | -0.02254 | 0.98 | baseline motor |  |
|  | -0.0008 | 0.0011 | 0.001356 | -0.00296 | 0.46 | motor slope |  |
|  | 0.0232 | 0.0639 | 0.148444 | -0.10204 | 0.72 | baseline parkinsonism |  |
|  | 0.0053 | 0.006 | 0.01706 | -0.00646 | 0.38 | parkinsonism slope |  |
| 1:207980901 | 0.0166 | 0.0163 | 0.048548 | -0.01535 | 0.31 | global AD pathology | rs4844620 |
|  | 0.004 | 0.0031 | 0.0156 | -0.00208 | 0.19 | amyloid deposition |  |
|  | 0.0314 | 0.0401 | 0.109996 | -0.0472 | 0.43 | tangle density |  |
|  | 1.13 |  | 0.95 | 1.34 | 0.17 | atherosclerosis |  |
|  | 0.92 |  | 0.77 | 1.1 | 0.35 | arteriol |  |
|  | 1.13 |  | 0.98 | 1.31 | 0.087 | dementia |  |
|  | -0.0989 | 0.0457 | -0.00933 | -0.18847 | 0.0306 | baseline global cognition |  |
|  | -0.0106 | 0.0039 | -0.00296 | -0.01824 | 0.0071 | global cognition slope |  |
|  | -0.0114 | 0.0101 | 0.008396 | -0.0312 | 0.26 | baseline motor |  |
|  | -0.0013 | 0.0009 | 0.000464 | -0.00306 | 0.18 | motor slope |  |
|  | 0.1112 | 0.0557 | 0.220372 | 0.002028 | 0.0459 | baseline parkinsonism |  |
|  | 0.0094 | 0.0054 | 0.019984 | -0.00118 | 0.071 | parkinsonism slope |  |
| 6:132467591 | 0.0134 | 0.0097 | 0.032412 | -0.00561 | 0.169 | global AD pathology | rs6925799 |
|  | -0.0087 | 0.0033 | -0.00223 | -0.01517 | 0.0075 | amyloid deposition |  |
|  | -0.0134 | 0.128 | 0.23748 | -0.26428 | 0.917 | tangle density |  |
|  | 0.78 |  | 0.42 | 1.44 | 0.425 | atherosclerosis |  |
|  | 0.81 |  | 0.45 | 1.47 | 0.492 | arteriol |  |
|  | 0.98 |  | 0.65 | 1.47 | 0.924 | dementia |  |
|  | -0.1231 | 0.1312 | 0.134052 | -0.38025 | 0.3485 | baseline global cognition |  |
|  | 0.0155 | 0.0114 | 0.037844 | -0.00684 | 0.1729 | global cognition slope |  |
|  | 0.0189 | 0.0309 | 0.079464 | -0.04166 | 0.54 | baseline motor |  |
|  | 0.0062 | 0.0028 | 0.011688 | 0.000712 | 0.0265 | motor slope |  |
|  | -0.1106 | 0.1674 | 0.217504 | -0.4387 | 0.5087 | baseline parkinsonism |  |
|  | -0.0014 | 0.0159 | 0.029764 | -0.03256 | 0.929 | parkinsonism slope |  |
| 12:76420006 | -0.0211 | 0.0186 | 0.015356 | -0.05756 | 0.26 | global AD pathology | rs73133134 |
|  | -0.0006 | 0.0035 | 0.00626 | -0.00746 | 0.86 | amyloid deposition |  |
|  | -0.1179 | 0.0455 | -0.02872 | -0.20708 | 0.00974 | tangle density |  |
|  | 1.11 |  | 0.91 | 1.35 | 0.287 | atherosclerosis |  |
|  | 0.82 |  | 0.67 | 1.01 | 0.0606 | arteriol |  |
|  | 0.89 |  | 0.75 | 1.05 | 0.1615 | dementia |  |
|  | 0.122 | 0.0532 | 0.226272 | 0.017728 | 0.0219 | baseline global cognition |  |
|  | 0.0103 | 0.0046 | 0.019316 | 0.001284 | 0.0261 | global cognition slope |  |
|  | -0.0106 | 0.0119 | 0.012724 | -0.03392 | 0.3727 | baseline motor |  |
|  | -0.0011 | 0.0011 | 0.001056 | -0.00326 | 0.32 | motor slope |  |
|  | 0.0592 | 0.0645 | 0.18562 | -0.06722 | 0.3588 | baseline parkinsonism |  |
|  | 0.0025 | 0.0062 | 0.014652 | -0.00965 | 0.6889 | parkinsonism slope |  |
| 19:56229834 | 0.0197 | 0.0201 | 0.059096 | -0.0197 | 0.33 | global AD pathology | rs77378270 |
|  | 0.0028 | 0.0038 | 0.010248 | -0.00465 | 0.453 | amyloid deposition |  |
|  | 0.0755 | 0.0494 | 0.172324 | -0.02132 | 0.1267 | tangle density |  |
|  | 1.28 |  | 1.04 | 1.58 | 0.0212 | atherosclerosis |  |
|  | 1.06 |  | 0.86 | 1.31 | 0.601 | arteriol |  |
|  | 1.07 |  | 0.84 | 1.21 | 0.94 | dementia |  |
|  | -0.0165 | 0.0581 | 0.097376 | -0.13038 | 0.7764 | baseline global cognition |  |
|  | -0.0083 | 0.0051 | 0.001696 | -0.0183 | 0.1005 | global cognition slope |  |
|  | 0.0043 | 0.0131 | 0.029976 | -0.02138 | 0.7427 | baseline motor |  |
|  | -0.0003 | 0.0012 | 0.002052 | -0.00265 | 0.8165 | motor slope |  |
|  | 0.0039 | 0.0702 | 0.141492 | -0.13369 | 0.956 | baseline parkinsonism |  |
|  | 0.0006 | 0.0067 | 0.013732 | -0.01253 | 0.9233 | parkinsonism slope |  |
| 2:175093901 | -0.0293 | 0.0158 | 0.001668 | -0.06027 | 0.0638 | global AD pathology | rs6752755 |
|  | -0.0045 | 0.003 | 0.00138 | -0.01038 | 0.126 | amyloid deposition |  |
|  | -0.05 | 0.0388 | 0.026048 | -0.12605 | 0.198 | tangle density |  |
|  | 0.91 |  | 0.76 | 1.07 | 0.247 | atherosclerosis |  |
|  | 0.8 |  | 0.67 | 0.95 | 0.0112 | arteriol |  |
|  | 0.94 |  | 0.82 | 1.08 | 0.3605 | dementia |  |
|  | 0.0726 | 0.0442 | 0.159232 | -0.01403 | 0.1007 | baseline global cognition |  |
|  | 0.0053 | 0.0038 | 0.012748 | -0.00215 | 0.1615 | global cognition slope |  |
|  | 0.0089 | 0.098 | 0.20098 | -0.18318 | 0.363 | baseline motor |  |
|  | 0 | 0.0009 | 0.001764 | -0.00176 | 0.934 | motor slope |  |
|  | -0.1074 | 0.0539 | -0.00176 | -0.21304 | 0.0464 | baseline parkinsonism |  |
|  | -0.0061 | 0.005 | 0.0037 | -0.0159 | 0.2272 | parkinsonism slope |  |
| 13:38206239 | 0.0173 | 0.0137 | 0.044152 | -0.00955 | 0.206 | global AD pathology | rs4943525 |
|  | 0.0029 | 0.0026 | 0.007996 | -0.0022 | 0.265 | amyloid deposition |  |
|  | 0.056 | 0.0337 | 0.122052 | -0.01005 | 0.0973 | tangle density |  |
|  | 1.1 |  | 0.95 | 1.27 | 0.194 | atherosclerosis |  |
|  | 0.99 |  | 0.86 | 1.15 | 0.912 | arteriol |  |
|  | 1.08 |  | 0.96 | 1.22 | 0.194 | dementia |  |
|  | -0.0794 | 0.0382 | -0.00453 | -0.15427 | 0.0377 | baseline global cognition |  |
|  | -0.0055 | 0.0033 | 0.000968 | -0.01197 | 0.0979 | global cognition slope |  |
|  | -0.0157 | 0.0085 | 0.00096 | -0.03236 | 0.065 | baseline motor |  |
|  | -0.0021 | 0.0008 | -0.00053 | -0.00367 | 0.0094 | motor slope |  |
|  | 0.0456 | 0.0468 | 0.137328 | -0.04613 | 0.3295 | baseline parkinsonism |  |
|  | 0.0082 | 0.0044 | 0.016824 | -0.00042 | 0.0624 | parkinsonism slope |  |
| 7:12137067 | 0.0964 | 0.1038 | 0.299848 | -0.10705 | 0.353 | global AD pathology | rs4721030 |
|  | 0.0904 | 0.0194 | 0.128424 | 0.052376 | 0.265 | amyloid deposition |  |
|  | 0.4341 | 0.2549 | 0.933704 | -0.0655 | 0.0888 | tangle density |  |
|  | 0.22 |  | 0.04 | 1.25 | 0.0879 | atherosclerosis |  |
|  | 0.72 |  | 0.22 | 2.32 | 0.5796 | arteriol |  |
|  | 1.63 |  | 0.73 | 3.64 | 0.234 | dementia |  |
|  | -0.5451 | 0.2496 | -0.05588 | -1.03432 | 0.0291 | baseline global cognition |  |
|  | -0.0083 | 0.0222 | 0.035212 | -0.05181 | 0.7102 | global cognition slope |  |
|  | -0.0549 | 0.0562 | 0.055252 | -0.16505 | 0.3289 | baseline motor |  |
|  | 0.004 | 0.0054 | 0.014584 | -0.00658 | 0.4587 | motor slope |  |
|  | -0.2242 | 0.3134 | 0.390064 | -0.83846 | 0.4745 | baseline parkinsonism |  |
|  | -0.0255 | 0.0311 | 0.035456 | -0.08646 | 0.413 | parkinsonism slope |  |

eTable 8. Relation of Cortical Protein Levels to Aging Phenotypes (for proteins measured in cortex, and for proteins which were related to an aging phenotype, at nominal significance)

| **Phenotype** | **Protein** | **beta or OR** | **SE of mean** | **p-value** | **LowerCL** | **UpperCL** |
| --- | --- | --- | --- | --- | --- | --- |
| global AD pathology | ACTR3B | -0.04469 | 0.01345 | 0.0009 | -0.07109 | -0.0183 |
| amyloid deposition | ACTR3B | -0.07645 | 0.02646 | 0.004 | -0.12838 | -0.02451 |
| tangle density | ACTR3B | -0.10159 | 0.02981 | 0.0007 | -0.1601 | -0.04308 |
| arteriol | ACTR3B | 0.995 |  | 0.862 | 1.149 | 0.9483 |
| dementia | ACTR3B | 0.745 |  | 0.00001 | 0.652 | 0.852 |
| baseline global cognition | ACTR3B | 0.1787 | 0.03993 | 0.00001 | 0.100437 | 0.256963 |
| cognition slope | ACTR3B | 0.01666 | 0.004097 | 0.00001 | 0.00863 | 0.02469 |
| baseline motor | ACTR3B | 0.01926 | 0.01024 | 0.06 | -0.00081 | 0.03933 |
| motor slope | ACTR3B | 0.000513 | 0.001219 | 0.6738 | -0.00188 | 0.002902 |
| baseline parkinsonism | ACTR3B | -0.1256 | 0.05004 | 0.0121 | -0.22368 | -0.02752 |
| parkinsonism slope | ACTR3B | -0.02091 | 0.005899 | 0.0004 | -0.03247 | -0.00935 |
| atherosclerosis | ACTR3B | 1.01 |  | 0.8874 | 0.876 | 1.165 |
| global AD pathology | EPHA7 | -0.02973 | 0.01351 | 0.028 | -0.05624 | -0.00322 |
| amyloid deposition | EPHA7 | -0.05545 | 0.02651 | 0.0368 | -0.10748 | -0.00341 |
| tangle density | EPHA7 | -0.06813 | 0.02993 | 0.0231 | -0.12689 | -0.00938 |
| arteriol | EPHA7 | 0.993 |  | 0.861 | 1.146 | 0.927 |
| atherosclerosis | EPHA7 | 1.115 |  | 0.1482 | 0.962 | 1.292 |
| dementia | EPHA7 | 1.026 |  | 0.687 | 0.904 | 1.166 |
| baseline global cognition | EPHA7 | -0.00591 | 0.03918 | 0.88 | -0.0827 | 0.070883 |
| cognition slope | EPHA7 | 0.003183 | 0.004023 | 0.4289 | -0.0047 | 0.011068 |
| baseline motor | EPHA7 | -0.00302 | 0.00988 | 0.76 | -0.02238 | 0.016345 |
| motor slope | EPHA7 | -0.00058 | 0.001214 | 0.6308 | -0.00296 | 0.001799 |
| baseline parkinsonism | EPHA7 | -0.01069 | 0.04814 | 0.8242 | -0.10504 | 0.083664 |
| parkinsonism slope | EPHA7 | -0.00469 | 0.005756 | 0.4152 | -0.01597 | 0.006592 |
| global AD pathology | LARP1 | -0.04843 | 0.01346 | 0.0003 | -0.07484 | -0.02202 |
| amyloid deposition | LARP1 | -0.06956 | 0.02655 | 0.009 | -0.12167 | -0.01745 |
| tangle density | LARP1 | -0.13486 | 0.02976 | 0.00001 | -0.19327 | -0.07644 |
| arteriol | LARP1 | 1.028 |  | 0.891 | 1.187 | 0.7011 |
| atherosclerosis | LARP1 | 1.098 |  | 0.203 | 0.951 | 1.268 |
| dementia | LARP1 | 0.643 |  | 0.00001 | 0.559 | 0.741 |
| baseline global cognition | LARP1 | 0.2393 | 0.03814 | 0.00001 | 0.164546 | 0.314054 |
| cognition slope | LARP1 | 0.02093 | 0.003839 | 0.00001 | 0.013406 | 0.028454 |
| baseline motor | LARP1 | 0.03409 | 0.009689 | 0.0004 | 0.0151 | 0.05308 |
| motor slope | LARP1 | 0.003136 | 0.001137 | 0.0058 | 0.000907 | 0.005365 |
| baseline parkinsonism | LARP1 | -0.177 | 0.0476 | 0.0002 | -0.2703 | -0.0837 |
| parkinsonism slope | LARP1 | -0.01939 | 0.005485 | 0.0004 | -0.03014 | -0.00864 |
| global AD pathology | MOXD1 | -0.01975 | 0.01399 | 0.1586 | -0.04721 | 0.00772 |
| amyloid deposition | MOXD1 | -0.05869 | 0.02768 | 0.0343 | -0.11303 | -0.00436 |
| tangle density | MOXD1 | -0.04287 | 0.03136 | 0.172 | -0.10442 | 0.01868 |
| arteriol | MOXD1 | 0.986 |  | 0.849 | 1.146 | 0.8546 |
| atherosclerosis | MOXD1 | 0.925 |  | 0.2995 | 0.797 | 1.072 |
| dementia | MOXD1 | 1.055 |  | 0.4225 | 0.925 | 1.204 |
| baseline global cognition | MOXD1 | -0.09825 | 0.04087 | 0.0163 | -0.17836 | -0.01814 |
| cognition slope | MOXD1 | -0.00598 | 0.004073 | 0.1424 | -0.01396 | 0.002003 |
| baseline motor | MOXD1 | -0.01723 | 0.01022 | 0.0919 | -0.03726 | 0.002801 |
| motor slope | MOXD1 | -0.00009 | 0.00116 | 0.938 | -0.00236 | 0.002184 |
| baseline parkinsonism | MOXD1 | 0.06665 | 0.04979 | 0.1808 | -0.03094 | 0.164238 |
| parkinsonism slope | MOXD1 | 0.002713 | 0.005588 | 0.6274 | -0.00824 | 0.013665 |
| global AD pathology | OLA1 | 0.02945 | 0.01359 | 0.0305 | 0.00278 | 0.05613 |
| amyloid deposition | OLA1 | 0.04836 | 0.02672 | 0.0707 | -0.00409 | 0.10081 |
| tangle density | OLA1 | 0.05883 | 0.03015 | 0.0514 | -0.00035 | 0.11801 |
| arteriol | OLA1 | 1.091 |  | 0.943 | 1.263 | 0.2423 |
| atherosclerosis | OLA1 | 1.147 |  | 0.0655 | 0.991 | 1.326 |
| dementia | OLA1 | 1.249 |  | 0.0008 | 1.097 | 1.422 |
| baseline global cognition | OLA1 | -0.09682 | 0.03985 | 0.0151 | -0.17493 | -0.01871 |
| cognition slope | OLA1 | -0.00966 | 0.004002 | 0.0158 | -0.0175 | -0.00182 |
| baseline motor | OLA1 | -0.00698 | 0.01001 | 0.4854 | -0.0266 | 0.01264 |
| motor slope | OLA1 | -0.00228 | 0.001158 | 0.0485 | -0.00455 | -1.03E-05 |
| baseline parkinsonism | OLA1 | -0.00119 | 0.04895 | 0.9806 | -0.09713 | 0.094752 |
| parkinsonism slope | OLA1 | 0.007228 | 0.005591 | 0.1962 | -0.00373 | 0.018186 |
| global AD pathology | STAU2 | -0.02385 | 0.0135 | 0.0776 | -0.05035 | 0.00264 |
| amyloid deposition | STAU2 | -0.00675 | 0.02657 | 0.7997 | -0.0589 | 0.04541 |
| tangle density | STAU2 | -0.06493 | 0.02992 | 0.0303 | -0.12366 | -0.00619 |
| arteriol | STAU2 | 1.041 |  | 0.9 | 1.204 | 0.5916 |
| atherosclerosis | STAU2 | 0.976 |  | 0.7343 | 0.846 | 1.125 |
| dementia | STAU2 | 0.793 |  | 0.0005 | 0.696 | 0.903 |
| baseline global cognition | STAU2 | 0.1283 | 0.03991 | 0.0013 | 0.050076 | 0.206524 |
| cognition slope | STAU2 | 0.01282 | 0.004042 | 0.0015 | 0.004898 | 0.020742 |
| baseline motor | STAU2 | 0.02592 | 0.01019 | 0.011 | 0.005948 | 0.045892 |
| motor slope | STAU2 | 0.002853 | 0.001196 | 0.0171 | 0.000509 | 0.005197 |
| baseline parkinsonism | STAU2 | -0.1838 | 0.04901 | 0.0002 | -0.27986 | -0.08774 |
| parkinsonism slope | STAU2 | -0.03076 | 0.005543 | 0.00001 | -0.04162 | -0.0199 |
| global AD pathology | THSD7A | 0.06671 | 0.01331 | 0.00001 | 0.04058 | 0.09284 |
| amyloid deposition | THSD7A | 0.14558 | 0.02606 | 0.00001 | 0.09442 | 0.19673 |
| tangle density | THSD7A | 0.12137 | 0.02967 | 0.00001 | 0.06314 | 0.1796 |
| arteriol | THSD7A | 0.948 |  | 0.822 | 1.095 | 0.4705 |
| atherosclerosis | THSD7A | 0.968 |  | 0.651 | 0.839 | 1.116 |
| dementia | THSD7A | 1.14 |  | 0.0455 | 1.003 | 1.295 |
| baseline global cognition | THSD7A | -0.1067 | 0.0398 | 0.0073 | -0.18471 | -0.02869 |
| cognition slope | THSD7A | -0.01306 | 0.003987 | 0.0011 | -0.02087 | -0.00525 |
| baseline motor | THSD7A | -0.00899 | 0.01004 | 0.3703 | -0.02867 | 0.010688 |
| motor slope | THSD7A | -0.00188 | 0.001169 | 0.1075 | -0.00417 | 0.000411 |
| baseline parkinsonism | THSD7A | 0.01444 | 0.04883 | 0.7675 | -0.08127 | 0.110147 |
| parkinsonism slope | THSD7A | -0.00072 | 0.005585 | 0.8968 | -0.01167 | 0.010227 |
| global AD pathology | TMEM106B | 0.01565 | 0.01385 | 0.259 | -0.01154 | 0.04283 |
| amyloid deposition | TMEM106B | 0.01704 | 0.02731 | 0.5327 | -0.03655 | 0.07064 |
| tangle density | TMEM106B | 0.06618 | 0.03075 | 0.0316 | 0.00583 | 0.12653 |
| arteriol | TMEM106B | 0.994 |  | 0.856 | 1.155 | 0.9414 |
| atherosclerosis | TMEM106B | 1.089 |  | 0.261 | 0.938 | 1.265 |
| dementia | TMEM106B | 1.273 |  | 0.0004 | 1.113 | 1.455 |
| baseline global cognition | TMEM106B | -0.1104 | 0.04062 | 0.0066 | -0.19002 | -0.03078 |
| cognition slope | TMEM106B | -0.00844 | 0.004101 | 0.0397 | -0.01648 | -0.0004 |
| baseline motor | TMEM106B | -0.0329 | 0.01015 | 0.0012 | -0.05279 | -0.01301 |
| motor slope | TMEM106B | -0.00489 | 0.001179 | 0.00001 | -0.0072 | -0.00258 |
| baseline parkinsonism | TMEM106B | 0.1261 | 0.04985 | 0.0114 | 0.028394 | 0.223806 |
| parkinsonism slope | TMEM106B | 0.02161 | 0.005688 | 0.0001 | 0.010462 | 0.032758 |
