# Supplementary material for: Genetic architecture of epigenetic cortical clock age in brain tissue from older individuals: alterations in CD46 and other loci
Source: Epigenetics. 2024 Aug 22;19(1):2392050. doi: 10.1080/15592294.2024.2392050 (PMC11346548; doi:10.1080/15592294.2024.2392050)
Supplement: figure_suppl.docx [file KEPI_A_2392050_SM6884.docx]

eFigure 1. Q-Q plots

**λ** =1.03

**λ=1.03**

ROSMAP and BDR GWAS meta-analysis

**λ=1.1**

ROSMAP GWAS
